# Covering the Combinatorial Design Space of Multiplex CRISPR/Cas Experiments in Plants

**DOI:** 10.3389/fpls.2022.907095

**Published:** 2022-06-20

**Authors:** Kirsten Van Huffel, Michiel Stock, Tom Ruttink, Bernard De Baets

**Affiliations:** ^1^Knowledge-based Systems (KERMIT), Department of Data Analysis and Mathematical Modelling, Faculty of Bioscience Engineering, Ghent University, Ghent, Belgium; ^2^Plant Sciences Unit, Flanders Research Institute for Agricultural, Fisheries and Food (ILVO), Melle, Belgium

**Keywords:** multiplex CRISPR/Cas screens, combinatorial gene knockout libraries, experimental design, plant genetic studies, crop breeding

## Abstract

Over the past years, CRISPR/Cas-mediated genome editing has revolutionized plant genetic studies and crop breeding. Specifically, due to its ability to simultaneously target multiple genes, the multiplex CRISPR/Cas system has emerged as a powerful technology for functional analysis of genetic pathways. As such, it holds great potential for application in plant systems to discover genetic interactions and to improve polygenic agronomic traits in crop breeding. However, optimal experimental design regarding *coverage* of the *combinatorial design space* in multiplex CRISPR/Cas screens remains largely unexplored. To contribute to well-informed experimental design of such screens in plants, we first establish a representation of the design space at different stages of a multiplex CRISPR/Cas experiment. We provide two independent computational approaches yielding insights into the *plant library size* guaranteeing full coverage of all relevant multiplex combinations of gene knockouts in a specific multiplex CRISPR/Cas screen. These frameworks take into account several design parameters (e.g., the number of target genes, the number of gRNAs designed per gene, and the number of elements in the combinatorial array) and efficiencies at subsequent stages of a multiplex CRISPR/Cas experiment (e.g., the distribution of gRNA/Cas delivery, gRNA-specific mutation efficiency, and knockout efficiency). With this work, we intend to raise awareness about the limitations regarding the number of target genes and order of genetic interaction that can be realistically analyzed in multiplex CRISPR/Cas experiments with a given number of plants. Finally, we establish guidelines for designing multiplex CRISPR/Cas experiments with an optimal coverage of the combinatorial design space at minimal plant library size.

## 1. Introduction

Genetic mutagenesis is a widespread and powerful strategy for the functional characterization of genes in various biological processes. It provides a complementary approach to the mapping of genotype-phenotype relationships based on quantitative genetic analyses [such as genome-wide association studies (Brachi et al., 2011) and quantitative trait locus mapping (Mauricio, 2001)], gene regulatory network analyses through differential expression (Clifton et al., 2006), and gene expression perturbation through RNA interference (Travella et al., 2006). Further, genetic mutagenesis may be used to test hypotheses of gene functional redundancy based on phylogenetic analyses of gene families (Zhang et al., 2018). Within this context, the Clustered Regularly Interspaced Short Palindromic Repeats (CRISPR)/Cas system has paved the way for targeted genome editing in many different organisms, including plant species (Brooks et al., 2014; Zhang et al., 2014, 2016; Ma et al., 2016; Fellmann et al., 2017; De Bruyn et al., 2020; Liu Z. et al., 2020). The CRISPR/Cas system relies on the delivery of a specific guide RNA (gRNA) and the Cas nuclease into a target cell. After formation of the gRNA/Cas complex, the gRNA directs Cas to induce DNA cleavage in the genomic sequence homologous to the gRNA. In case the target sequence is located inside a conserved region, e.g., in highly homologous paralogous genes, multiple sites in the genome can be cleaved in parallel. Subsequently, each cleaved site can undergo error-prone DNA repair *via* the non-homologous end-joining pathway, creating a mutation. Mutations in the coding region of a gene might disrupt the open reading frame or one of the mRNA splice sites, leading to the formation of a mutated, truncated, or out-of-frame protein sequence that, in turn, can result in the knockout (KO) of gene function (Mali et al., 2013). If the gRNA was designed to target regulatory sequences of the gene, gene expression levels may be disrupted. Ultimately, examining the impact of these genetic perturbations on the plant phenotype can contribute to unraveling gene function and genetically improving agronomic traits in breeding materials.

The multiplex CRISPR/Cas system forms a relatively novel extension to the standard CRISPR/Cas system (Shen J. P. et al., 2017; Zhou et al., 2020), allowing for the simultaneous editing of multiple unique targets in a single plant cell (Zhang et al., 2016). The latter enables to identify genetic redundancy as well as genetic interactions, which contributes to elucidating the complex interplay of genes in metabolic and/or regulatory pathways. Thus, multiplex CRISPR/Cas genome editing speeds up the functional analysis of genetic pathways thanks to its ability to specifically target multiple genes simultaneously. To accomplish simultaneous editing activity, multiple gRNA/Cas complexes are co-expressed in each target cell. A first strategy to deliver multiple gRNA sequences per cell is to assemble multiplex gRNA/Cas constructs by cloning methods such as Golden Gate ligation (Engler et al., 2008; Ma et al., 2015) and Gibson Assembly (Jacobs et al., 2017), yielding binary vectors with arrays of gRNA expression cassettes for stable *Agrobacterium* transformation. Second, multiple vectors transiently expressing Cas and/or one or more gRNAs can be co-transfected into protoplasts *via* electroporation or polyethylene glycol-mediated transfection, followed by whole plant regeneration (Toda et al., 2019). Third, preassembled gRNA/Cas ribonucleoprotein complexes with mixtures of gRNAs can be delivered into plant cells *via* particle bombardment (Liang et al., 2018), polyethylene glycol-mediated transfection, or nanoparticles (Cunningham et al., 2018).

**Figure 1:**
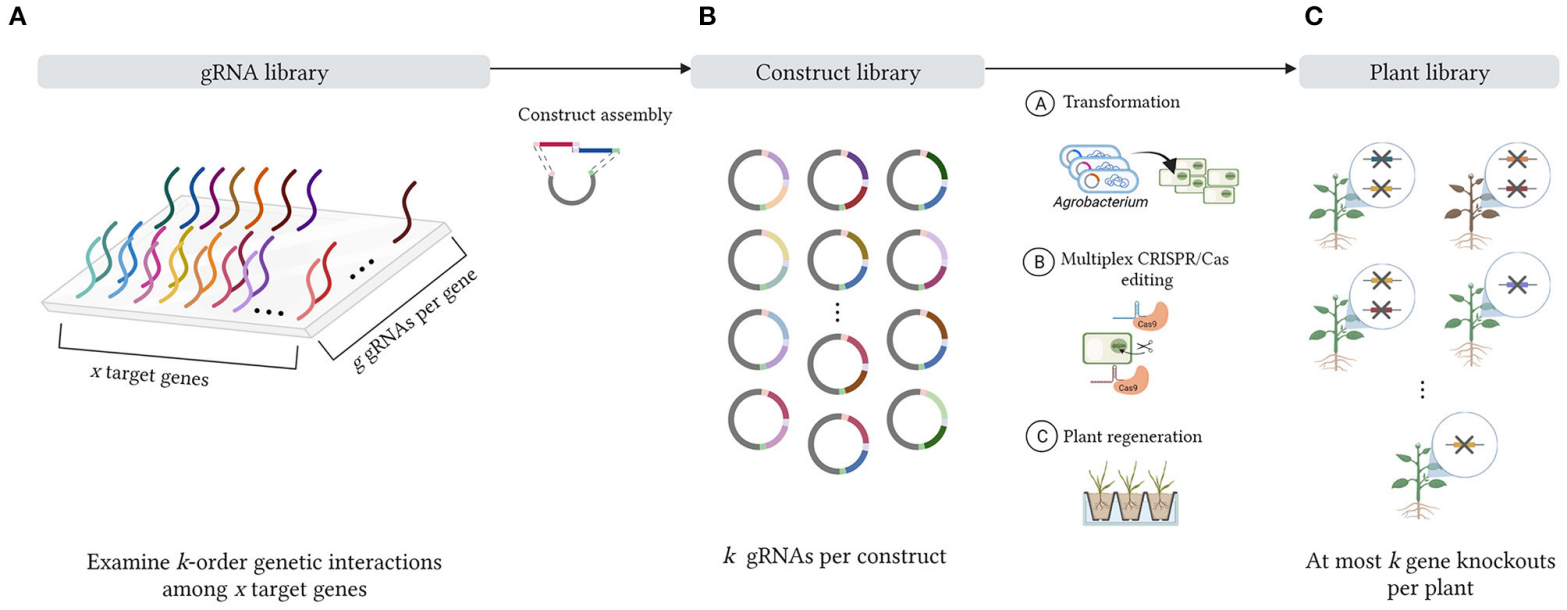
Schematic representation of a multiplex CRISPR/Cas experiment. **(A)** First, *x* target genes are selected for CRISPR/Cas editing. A number of *g* gRNAs are designed for each target gene, forming an initial gRNA library. **(B)** During vector assembly, gRNA sequences from the gRNA pool are randomly combined into a combinatorial gRNA/Cas construct library with *k* gRNA sequences per vector to study the effects of *k*-combinations of gene knockouts, i.e., *k*-order genetic interactions among the set of *x* target genes. After transformation of the combinatorial gRNA/Cas constructs into the target cells, the gRNA/Cas complexes can be expressed, leading to genome editing of the associated target loci. **(C)** After plant regeneration, a diverse mutant plant collection is obtained with at most *k* gene knockouts per plant, forming the plant library.

Each of the aforementioned gRNA delivery methods can be designed to introduce a specific number of gRNAs per target cell, hence enabling the study of a particular order of interaction among a set of target genes in a multiplex CRISPR/Cas system. For simplicity, we focus on one prototypical delivery method, namely the stable transformation of multiplex gRNA/Cas constructs containing an array of gRNA expression cassettes into target cells. This approach starts with the design of a pool of gRNA sequences targeting a set of target genes (Figure 1A), after which these gRNA sequences are randomly assembled into combinatorial gRNA/Cas constructs with a specific number of gRNA sequences per vector (Figure 1B). For instance, to study pairwise interactions of genes, combinatorial gRNA/Cas constructs with two gRNAs per vector can be produced, such that, after transformation, two gRNA/Cas complexes are generated per plant cell. Likewise, for investigating up to *k*-order genetic interactions, combinatorial gRNA/Cas constructs are designed so that *k* gRNA/Cas complexes are expressed in each target cell. Throughout this paper, the term *construct library* will refer to the collection of all combinatorial gRNA/Cas constructs that can be generated from the initial gRNA pool by randomly sampling *k* gRNAs (with replacement) into an expression array. After transformation of the combinatorial gRNA/Cas construct library into the target cells (one construct per independent cell), the corresponding combinatorial gRNA/Cas activity results in a collection of cells containing different combinations of gene knockouts. Following plant regeneration of a random selection of these mutated cells, a genetically diverse collection of mutated plants is obtained, which is referred to as the *plant library* (Figure 1C). All genotypes that can theoretically be present in the *plant library*, i.e., all possible combinations of *k* (or fewer) target gene knockouts, constitute the *plant design space*. We gradually build up and visualize this design space in Section 2.1. By subjecting the *plant library* to a phenotypic screen (e.g., one that examines variations in traits such as flowering time, leaf density, internode length, number of root nodules, maturity time, metabolic profile, plant height or drought tolerance), one can assess the effect of many combinations of gene knockouts on the plant phenotype in parallel. In this manner, a deeper understanding of the genetic interactions in a specific metabolic or regulatory pathway can be acquired. Recent studies illustrate how insights gained from these assays have contributed to improving several agronomic traits in crop breeding (Li et al., 2018; Zhang Y. et al., 2020).

As a multiplex CRISPR/Cas experiment is designed to examine a larger number of target genes (*x*) and order of genetic interaction (*k*), the number of possible *k*-combinations of gene knockouts that occur in the *plant design space*, denoted by (xk), expands quickly. Accordingly, there is a combinatorial explosion of the number of plants that need to be screened in order to study all *k*-order genetic interactions. The (combinatorial) *coverage* (γ_*x,k*_) of a plant library is defined as the fraction of all (xk) gene knockout combinations that is contained at least once in this plant library. The plant library size required for full coverage refers to the minimal number of plants that needs to be included into a plant library to completely cover all (xk) gene knockout combinations in a multiplex CRISPR/Cas experiment (reaching γ_*x,k*_ = 1) and is denoted by *N*_*x,k*_. Note that in our model representation the plant library is obtained by random selection and regeneration of mutated cells. Due to the stochastic nature of this sampling process, *N*_*x,k*_ is a stochastic variable as well, its value varying with every execution of a specific multiplex CRISPR/Cas experiment. Quantification of the expected value and the standard deviation of *N*_*x,k*_ for a given experiment is a main objective of this study. The central parameters and variables in our study are summarized in Table 1.

**Table 1:** Central design parameters and stochastic variables of this study.

**Symbol**	**Short description**
**Design parameters**	
*x*	The number of target genes in a multiplex CRISPR/Cas experiment
*k*	The order of genetic interaction among the target genes to be investigated
*N*	The number of plants in a plant library, also called the *plant library size*
(xk)	The number of possible *k*-combinations of gene knockouts for *x* target genes
**Stochastic variables**	
γ_*x,k*_	The fraction of all (xk) gene knockout combinations that is contained by a plant library, also called
	the *(combinatorial) coverage* of a plant library
*N*_*x,k*_	The minimal plant library size required for full coverage (γ_*x,k*_ = 1)

Gaining insight in *N*_*x,k*_ for a multiplex CRISPR/Cas experiment is vital. Consider performing such an experiment to investigate all *k*-combinations of gene knockouts for a set of *x* target genes. Assume that the final plant library size *N* is too small to contain all (xk) gene knockout combinations (*N*< *N*_*x,k*_), hence not reaching full coverage (γ_*x,k*_ < 1). Performing a phenotypic screen on this plant library might give rise to misleading conclusions in two ways. First, effective combinations of gene knockouts might be misclassified as not associated with the desired phenotype as a result of not being represented in the plant library, leading to false-negative results. Second, one cannot evaluate whether the absence of a particular combination of gene knockouts in the plant library is due to lethal effects or rather the result of an inadequate plant library size. Therefore, a main consideration when determining the size *N* of the plant library resulting from a multiplex CRISPR/Cas experiment is to account for coverage of all (xk) gene knockout combinations. However, optimal design of such experiments in plants has remained largely unexplored in this regard. Existing tools that assist in determining an appropriate sample size for multiplex CRISPR/Cas screens in mammalian cells are not applicable to screens in plants due to different experimental protocols. Additionally, focus on the exploration of the complete combinatorial design space is lacking in these studies (Nagy and Kampmann, 2017; Shen J. P. et al., 2017; Imkeller et al., 2020; Diehl et al., 2021).

In this study, first and foremost, we suggest two independent approaches for determining *N*_*x,k*_ for multiplex CRISPR/Cas experiments in plants. The first approach makes use of computational simulations, reproducing subsequent stages of a multiplex CRISPR/Cas experiment *in silico*, to gain insight into *N*_*x,k*_. The second approach employs the BioCCP framework, which was presented in our previous work (Van Huffel et al., 2022), to provide a quick approximation of *N*_*x,k*_ and related statistics. BioCCP is a general framework based on the Coupon Collector Problem (CCP) studied in probability theory and statistics. The CCP allows one to determine minimal sample sizes for screening experiments in combinatorial biotechnology that guarantee full coverage of the design space. Apart from establishing the computation of *N*_*x,k*_ using these frameworks, we illustrate how *N*_*x,k*_ is impacted by some critical design parameters of a multiplex CRISPR/Cas experiment (e.g., the number of target genes, the number of elements in the combinatorial gRNA array, the relative abundances of gRNAs in the combinatorial gRNA/Cas construct library, the guide-specific genome editing efficiency and the global knockout efficiency). By means of a quantitative analysis, we demonstrate that a naive approach for experimental design might become prohibitively expensive, and that the maximal number of plants that can be genotyped and phenotyped in a multiplex CRISPR/Cas screen imposes limitations on the number of target genes and order of genetic interaction that can be investigated. Finally, we propose two main strategies (named the *Split–Select–Combine* strategy and the *Overshoot–Select–Purify* strategy) for designing multiplex CRISPR/Cas experiments with a minimal *N*_*x,k*_, and establish additional guidelines for experimental design to optimize design space coverage at minimal plant library size.

## 2. Results

### 2.1. Representation of the Design Space and Stochastic Sampling in a Multiplex CRISPR/Cas Experiment

**Figure 2:**
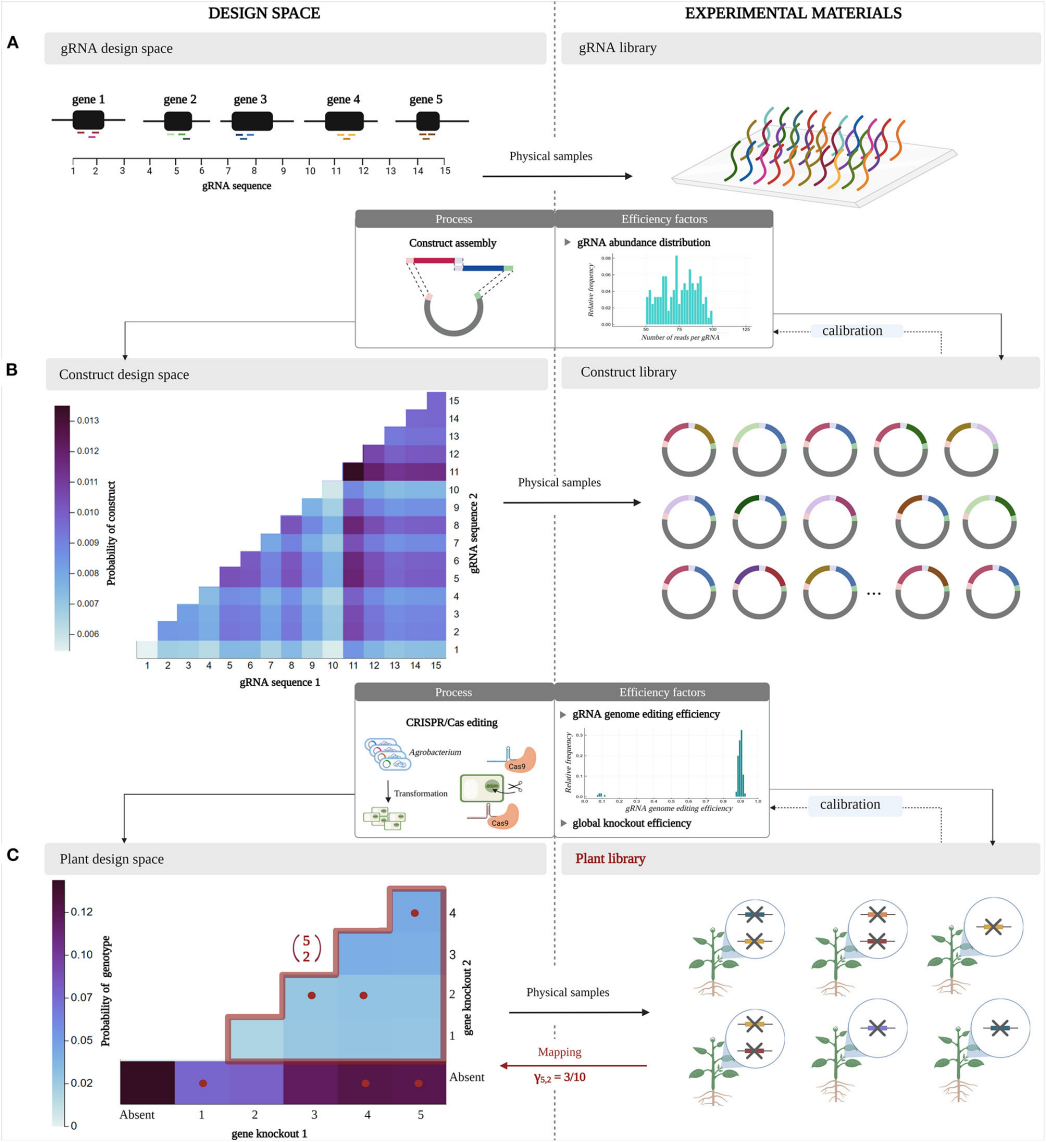
Design space representation and parallel experimental materials at different stages in a multiplex CRISPR/Cas experiment. A *design space* at a certain point in the experiment is considered as a virtual space encompassing all possible designs, whereas a *library* represents a collection of physical samples from the design space. These material experimental units are obtained by random sampling of the design space according to a sampling distribution, which describes the probability of sampling each design and is determined by inefficiencies at different stages of the multiplex CRISPR/Cas protocol. **(A)** Here, an experiment targeting pairwise combinations of five target genes is considered. Three gRNAs are designed per gene. **(B)** In the construct library, two gRNAs are included per gRNA/Cas construct. **(C)** The resulting plant library contains up to two gene knockouts per plant.

In the following, we discuss the representation of the design space at different stages of a multiplex CRISPR/Cas experiment. Additionally, we clarify how experimental materials created in such an experiment can be interpreted as physical samples from a virtual design space. These ideas are depicted in Figure 2.

Consider a multiplex CRISPR/Cas experiment that aims to investigate pairwise interactions among a set of five target genes. Such an experiment departs from an initial pool of gRNA sequences that are specifically designed to target this set of genes (three gRNAs per gene). At this stage, the *design space* encompasses all possible gRNA sequences (Figure 2A). In the following, we regard a *library* as the collection of experimental units that represent material samples from the *design space*. Here, the *gRNA library* is the collection of gRNA sequences that are generated in the laboratory, as a physical equivalent of the virtual *gRNA design space*.

The combinatorial aspect of the multiplex CRISPR/Cas experiment arises as these gRNA sequences are randomly combined into gRNA/Cas constructs through vector assembly. In Figure 2B, each construct contains a random combination of two gRNA sequences. All possible combinations of two gRNAs that can occur in these constructs form the design space at the construct level, called the *construct design space* (Figure 2B). During a multiplex CRISPR/Cas experiment, gRNA/Cas constructs containing random combinations of gRNAs are randomly collected, forming a *construct library*. Importantly, not all constructs (i.e., gRNA combinations) are equally likely to be sampled, but occur with different relative abundances (due to biases during synthesis of gRNA sequences, quantification, and non-equal pooling during assembly of the constructs). Therefore, the sampling process takes place according to a sampling distribution, shaped by the probability of sampling each gRNA combination from the *construct design space*. We assume that the probability of encountering a specific pairwise combination of gRNA sequences in a gRNA/Cas construct equals the product of the probabilities of the individual gRNA sequences occurring in a construct, i.e., these occurrences are independent.

Continuing to the next stage of the experiment, the *construct library* is delivered into target plant cells by *Agrobacterium* transformation, assuming the integration of one construct per cell. In these target cells, expression of the gRNA/Cas complexes leads to genome editing with a gRNA-specific efficiency. A specific fraction of the induced mutations will lead to an effective knockout of gene function. The *design space* at this stage is the collection of all plant genotypes that can occur, referred to as the *plant design space* (Figure 2C). In the example of Figure 2C, we distinguish three classes of genotypes: those characterized by a pairwise combination of gene knockouts, a single knockout, or the absence of knockouts. Not every combination of knockouts has the same probability of being sampled from the design space, hence does not occur with the same frequency in the mutated *plant library*. The latter is caused by three factors: (1) the unequal relative abundances of gRNAs in the *construct library*, (2) the varying levels of genome editing activity across the set of gRNAs, and (3) the varying impact of a given mutation at a given location of the gene on the function and activity of the encoded protein (not every mutation results in an effective gene knockout). Random selection of mutated cells for plant regeneration in order to construct a *plant library* implies sampling combinations of gene knockouts according to a sampling distribution that integrates these three inefficiencies at the gRNA level for every target gene combination. Here, once more we apply an independence assumption, considering the probability of a pairwise combination of gene knockouts appearing in a plant cell to be equal to the product of the probabilities of occurrence of the individual gene knockouts in a plant cell.

In the remainder of this paper, we will focus on the exploration of the *plant design space*. The multiplex CRISPR/Cas experiment depicted in Figure 2 aims to study all pairwise combinations of gene knockouts for five target genes. Here, the total number of possible pairwise combinations is denoted by (52). The central study aim here is to quantify the number of plants that is to be randomly collected to encounter all (52) possible combinations of gene knockouts in the *plant library*; this number is denoted by *N*_5, 2_. This way, the phenotypic effect of all 2-order genetic interactions among the set of five target genes can be assessed. Recall that the *coverage* γ_5, 2_ is defined as the fraction of all (52) pairwise gene knockout combinations that is included in a *plant library* of a given size. For the CRISPR/Cas experiment visualized in Figure 2, the gene knockout combinations present in the plant library are mapped back to the plant design space (Figure 2C). It can be seen that by randomly sampling six plants, in this case three out of the 10 possible pairwise combinations of gene knockouts are present, resulting in a coverage γ_5, 2_ of 0.3. The other three plants are not counted as they, by chance, only contain a single knockout and are considered a by-product in the plant library.

### 2.2. Computing the Plant Library Size That Guarantees Full Combinatorial Coverage During Screening

First, we outline a simulation-based approach for computing *N*_*x,k*_ of a multiplex CRISPR/Cas screen in plants. More specifically, we describe how to statistically sample the design spaces described in Section 2.1, taking into account the efficiencies at subsequent stages of the experiment. Recall that *N*_*x,k*_ of a multiplex CRISPR/Cas screen is defined as the minimal size of the plant library or number of plants to be screened in order to encounter all (xk) combinations of gene knockouts in the set of genotypes at least once, such that the effect of all relevant genetic interactions on the phenotype can be explored. As a basis for comparison, we also compute the minimal plant library size for full coverage of single gene knockouts in a (non-multiplex) CRISPR/Cas experiment, denoted by *N*_*x*,1_, before considering coverage of pairwise and triple gene knockout combinations in multiplex CRISPR/Cas experiments. Secondly, an alternative approach of computing *N*_*x,k*_ and some related statistics is presented through the use of our BioCCP.jl package (Van Huffel et al., 2022). Both approaches are available on GitHub (https://github.com/kirstvh/MultiplexCrisprDOE), enabling researchers to compute *N*_*x,k*_ and related statistics for custom multiplex CRISPR/Cas experiments.

#### 2.2.1. A Simulation-Based Approach to Compute the Expected Value and Standard Deviation

In the following paragraphs, we describe a simulation experiment to determine *N*_*x,k*_ of a multiplex CRISPR/Cas screen that aims to study the *k*-order genetic interactions among a set of *x* target genes. In this simulation, we generate plant genotypes *in silico* by modeling the subsequent stages of a multiplex CRISPR/Cas experiment (Jacobs et al., 2017). The genotype of each plant is represented by its set of gene knockouts. We virtually collect a set of these plants by random sampling, while storing the relevant combinations of gene knockouts observed in each plant. The process of collecting plants is halted as all (xk) combinations of gene knockouts are represented, guaranteeing the study of all *k*-order genetic interactions. This experiment is repeated 500 times to obtain an estimate of the expected value (E[*N*_*x,k*_]) and associated standard deviation (σ[*N*_*x,k*_]) of the number of plants required for full coverage.

**Figure 3:**
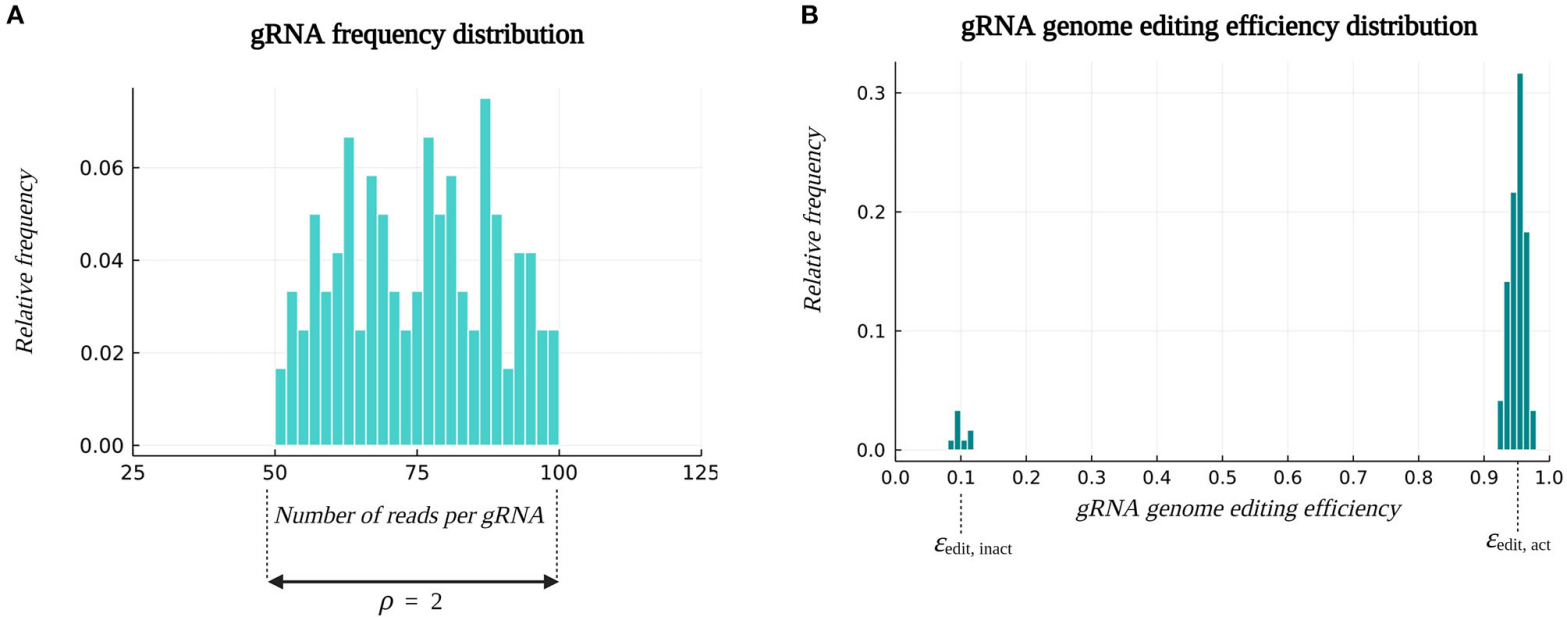
Example of default distributions. **(A)** The gRNA frequency distribution in the construct library. Parameter ρ denotes the ratio of the frequency of the most abundant gRNA to the frequency of the least abundant gRNA, and is by default set at the value of 2. **(B)** The gRNA genome editing efficiency distribution. By default, 90% of the gRNAs are active, having an average genome editing efficiency ϵ_edit, act_ of 0.95, and 10% of the gRNAs are inactive, characterized by an average genome editing efficiency ϵ_edit, inact_ of 0.1. Information on the construction of these distributions and the sampling process can be found in Sections 4.1.1 and 4.1.2.

Consider a multiplex CRISPR/Cas experiment targeting *x* = 20 genes. For each target gene, *g* = 6 different gRNAs are designed, which results in a pooled library with a total number of 120 gRNAs, reflecting the typical diversity feasible to clone in parallel *via* Golden Gate ligation and Gibson Assembly (Jacobs et al., 2017; Bai et al., 2020). Each gRNA is assumed to target only one locus in the genome. From this initial gRNA pool, multiplex gRNA/Cas constructs are assembled, sampling *r* gRNA sequences per construct. In this case, *r* equals *k*, since the goal is to study *k*-order genetic interactions among the target genes (Table 2). In an ideal gRNA/Cas construct library, all gRNAs are represented with the same frequencies. However, due to inaccuracies and technical constraints during gRNA synthesis, quantification and vector assembly steps, the abundance of gRNAs over the constructs is not uniform. We describe the gRNA abundance distribution in the construct library by the ratio of the frequency of the most abundant gRNA to the frequency of the least abundant gRNA, symbolized by ρ. More information on the construction of this distribution can be found in Section 4.1.1. The relative frequencies of the gRNAs are taken into account when generating the construct library *in silico*. Figure 3A depicts the gRNA abundance distribution (also called the frequency distribution) used for illustration throughout this study.

**Table 2:** Input parameters and outcome variables of the multiplex CRISPR/Cas simulation experiment.

**Symbol**	**Short description**	**Default value**
	** Input parameters**	
*x*	The number of target genes	20
*g*	The number of gRNAs designed per target gene	6
*k*	The order of genetic interaction to investigate	
	Single gene knockouts	1
	Pairwise combinations of gene knockouts	2
	Triple combinations of gene knockouts	3
*r*	The number of gRNAs per gRNA/Cas construct	*k*
ρ	The ratio of the frequency of the most abundant gRNA to the frequency of the least abundant gRNA
	in the gRNA/Cas construct library	2
*f_act_*	The fraction of total number of gRNAs that are active	0.9
*ϵ_edit, act_*	The average genome editing efficiency for active gRNAs	0.95
*ϵ_edit, inact_*	The average genome editing efficiency for inactive gRNAs	0.1
*ϵ_KO_*	The global knockout efficiency	0.8
	** Outcome variables**	
E[*N*_*x,k*_]	The expected value of the plant library size for full coverage of all *k*-combinations	
	of gene knockouts for a set of *x* target genes	
σ[*N*_*x,k*_]	The standard deviation of the plant library size for full coverage of all *k*-combinations	
	of gene knockouts for a set of *x* target genes	

Following the simulation of vector assembly, the gRNA/Cas constructs are virtually transformed into plant cells, assuming one construct per plant cell. The corresponding *k* gRNA/Cas complexes are assumed to induce mutations in the associated target loci, potentially resulting in a successful gene knockout. Importantly, not every gRNA/Cas complex brings about a mutation and not every mutation results in a loss-of-function gene knockout. Hence, at most *k* gene knockouts per plant genotype can be achieved. In this context, a genome editing efficiency ϵ_edit_ specific to each gRNA and a global knockout efficiency ϵ_KO_ is introduced. The genome editing efficiency ϵ_edit_ of a gRNA indicates the relative frequency by which a gRNA accomplishes a genome edit in the target sequence. A distinction is made between a group of active gRNAs with a high average genome editing efficiency ϵ_edit, act_ = 0.95 and a group of inactive gRNAs with a low average genome editing efficiency ϵ_edit, inact_ = 0.1. The fraction *f*_act_ of all gRNAs that is assumed to be active, i.e., the fraction of gRNAs following a distribution with a high average genome editing efficiency, was set at 0.9 (Table 2). Given the fraction of active gRNAs and the average editing efficiency of active and inactive gRNAs, a probability distribution for the genome editing efficiency is constructed from which a genome editing efficiency is sampled for each gRNA. More information on the construction of this distribution can be found in Section 4.1.2. An example distribution is represented in Figure 3B. The global knockout efficiency ϵ_KO_ indicates the fraction of genome edits leading to a loss-of-function gene knockout. By default, the value of ϵ_KO_ is set to 0.8.

After executing the aforementioned stages, a plant genotype with a specific combination of (at most *k*) gene knockouts is obtained. Plants are collected until all (xk) combinations of gene knockouts are seen, i.e., *N*_20,*k*_ is reached. Note that during this simulation, several (in)efficiencies are taken into account at subsequent steps of the experiment: (1) the imbalance in the abundances of gRNAs in the gRNA/Cas construct library, (2) the genome editing efficiency of each gRNA when inducing mutations in the plant genomes, and (3) the global knockout efficiency indicating the fraction of mutations leading to loss-of-function of the gene product. Section 2.2.3 explains how these efficiency parameters can be inferred from real experimental data.

**Figure 4:**
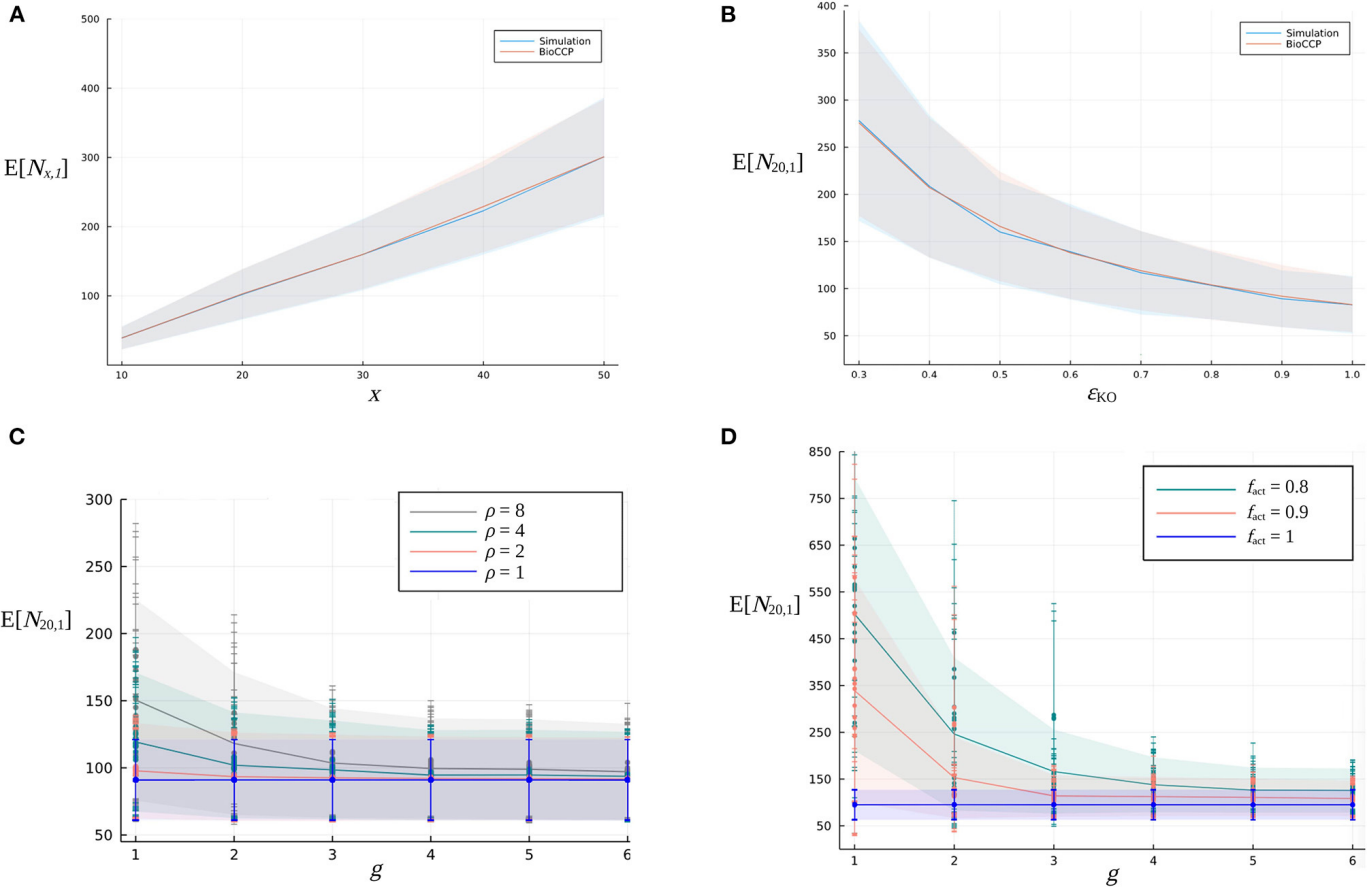
Relation between the expected value of the minimal plant library size for full coverage of all single gene knockouts and design parameters of a multiplex CRISPR/Cas experiment. **(A)** Effect of an increasing number of *x* target genes on E[*N*_*x*,1_]. **(B)** Effect of the global knockout efficiency ϵ_KO_ on E[*N*_20,1_]. The blue curve indicates the simulation-based results, while the red curve indicates the BioCCP-based results. The width of the shaded area around these curves represents σ[*N*_20,1_]. In each of the graphs, the parameter under investigation is varied while the values of the other parameters are fixed at the default values of the CRISPR/Cas experiment as specified in Table 2. **(C)** Effect of parameter ρ, a measure for the width of the gRNA frequency distribution, for an increasing number of *g* gRNAs per gene on E[*N*_20,1_]. **(D)** Effect of the fraction *f*_act_ of active gRNAs for an increasing number of *g* gRNAs per gene on E[*N*_20,1_].

**Figure 5:**
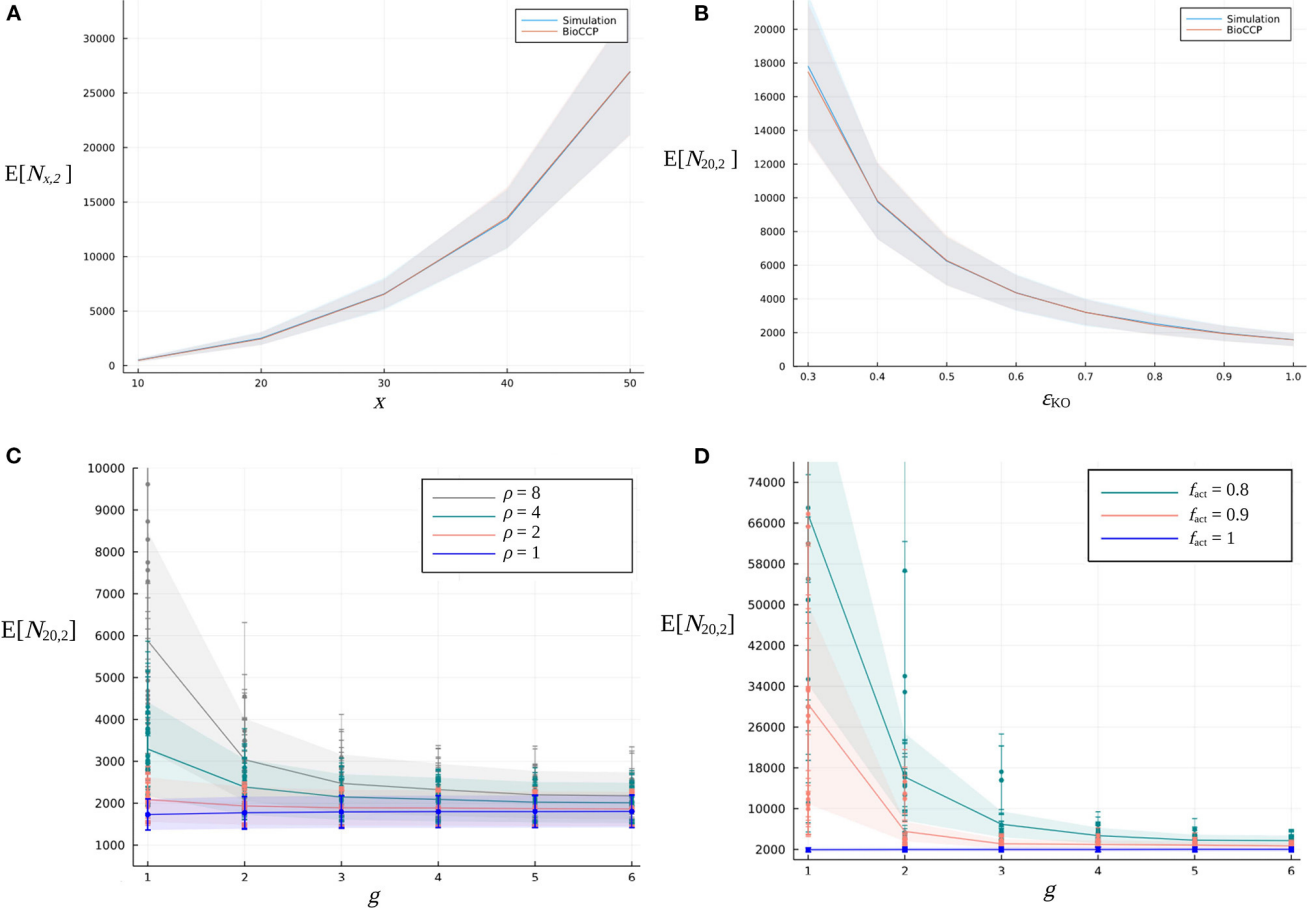
Relation between the expected value of the plant library size for full coverage of pairwise combinations of gene knockouts and design parameters of a multiplex CRISPR/Cas experiment. **(A)** Effect of an increasing number of *x* target genes on E[*N*_*x*,2_]. **(B)** Effect of the global knockout efficiency ϵ_KO_ on E[*N*_20,2_]. The blue curve indicates the simulation-based results, while the red curve indicates the BioCCP-based results. The width of the shaded area around these curves represents σ[*N*_20,2_]. In each of the graphs, the parameter under investigation is varied while the values of the other parameters are fixed at the default values of the CRISPR/Cas experiment as specified in Table 2. **(C)** Combined effect of parameter ρ of the gRNA frequency distribution for an increasing number of *g* gRNAs per gene on E[*N*_20,2_]. **(D)** Effect of the fraction *f*_act_ of active gRNAs for an increasing number of *g* gRNAs per gene on E[*N*_20,2_].

Multiplex CRISPR/Cas simulation experiments were executed following the procedure described above in order to compute the expected value and standard deviation of *N*_20,1_, *N*_20,2_, and *N*_20,3_. In these simulations, default settings of the input parameters were employed as specified in Table 2. The results are summarized in Table 3. Next, the influence of experimental parameters on *N*_*x*,1_ and *N*_*x*,2_ was investigated (Figures 4, 5). For this purpose, we perform CRISPR/Cas simulation experiments, employing the default settings in Table 2 for all parameters, except for the parameter under investigation.

- Firstly, we vary the number of target genes *x* in the CRISPR/Cas experiment in the range [10, 50]. Figure 4A illustrates that, for this specific range of values for *x*, the expected value of *N*_*x*,1_ increases in an approximately linear way. Figure 5A visualizes how *N*_*x*,2_ escalates quickly with an increasing number of *x* target genes, due to the total number of (x2) pairwise combinations and the corresponding combinatorial plant design space expanding combinatorially with a larger *x*.
- Figures 4, 5B demonstrate the impact of the global knockout efficiency ϵ_KO_ on *N*_*x,k*_. As expected, less effective gene editing inevitably demands a larger plant library size to completely cover all single gene knockouts (*N*_20,1_) as well as pairwise combinations of gene knockouts (*N*_20,2_).
- In Figures 4, 5C, the combined effect of the parameter ρ of the gRNA frequency distribution and the number of gRNAs per gene on *N*_20,1_ and *N*_20,2_ is illustrated. For a fixed number of *g* gRNAs per gene, it is clear that a more uneven gRNA frequency distribution, indicated by a larger ratio ρ, substantially increases the plant library size *N*_*x,k*_ for full coverage. Importantly, when more gRNAs are designed per gene, the increase of E[*N*_20,*k*_] caused by a higher ρ gradually diminishes. The latter suggests that, in the case of a construct library with highly underrepresented gRNA sequences, the plant library size for full coverage might be reduced by including more gRNAs per gene in the experiment. In Figures 4, 5C, the genome editing efficiency of all gRNAs is set at the ideal value of 1 (instead of sampling them from the genome editing efficiency distribution) in order to isolate the effect of sampling the gRNA relative frequencies from distributions with different ρ. Note that for each value of ρ, the set of gRNA relative frequencies was sampled multiple times from the same distribution, resulting in a variable outcome for E[*N*_20,*k*_] at a specific ρ (represented by different data points in the graph).
- In Figures 4, 5D, the fraction *f*_act_ of active gRNAs is demonstrated to exhibit a similar type of relation to *N*_*x,k*_. Here, several series of genome editing efficiencies were drawn from genome editing distributions characterized by a specific value of *f*_act_. The expected value of *N*_20,*k*_ of each corresponding multiplex CRISPR/Cas experiment is represented by a different data point in the graph. Clearly, at a fixed number of *g* gRNAs per gene, CRISPR/Cas experiments with a lower *f*_act_ require a higher number of plants for full coverage. Yet, this increasing effect on *N*_*x,k*_ can again be mitigated by including a greater number of gRNAs per gene. Note that the experiments visualized in Figures 4, 5D are executed by applying a uniform gRNA frequency distribution.

**Table 3:** Statistics of *N*_20,1_, *N*_20,2_, and *N*_20,3_ resulting from the simulation- and BioCCP-based approaches for the multiplex CRISPR/Cas experiments as specified by the default parameters in Table 2.

	**Simulation**			**BioCCP**		
***k***	**E[*N*_20,*k*_]**	**σ[*N*_20,*k*_]**	**Runtime**	**E[*N*_20,*k*_]**	**σ[*N*_20,*k*_]**	**Runtime**
1	103 plants	35 plants	0.2752 s	104 plants	37 plants	0.0015 s
2	2,518 plants	581 plants	6.503 s	2,453 plants	580 plants	0.0272 s
3	32,154 plants	5,825 plants	302.9 s	31,348 plants	5,979 plants	1.839 s

*Each simulation experiment is repeated 500 times*.

Figures 4, 5 demonstrate that *N*_20,2_ is in general more sensitive to changes of the values of design parameters as compared to *N*_20,1_. For graphs visualizing the impact of experimental design parameters on *N*_20,3_, we refer to the Supplementary Figure 1.

#### 2.2.2. BioCCP-Based Approach to Compute the Expected Value and Standard Deviation and Other Relevant Statistics

In previous work, we designed the BioCCP.jl package in the Julia Programming Language (Bezanson et al., 2017), providing tools for computing a minimal sample size that adequately covers the design space of combinatorial screening experiments in biotechnology (available at https://github.com/kirstvh/BioCCP.jl). This package reformulates the computation of a minimal sample size for covering the design space of a combinatorial library as a variant of the Coupon Collector Problem (CCP), a well-known problem in probability theory and statistics (Flajolet et al., 1992; Doumas and Papanicolaou, 2016). The standard formulation of the CCP describes a situation where there are *n* different types of “coupons” of which a collector tries to obtain a complete set (e.g., a set of stickers). Therefore, (s)he samples repeatedly with replacement one coupon at a time from a population (e.g., cereal boxes that each contain one random sticker). The goal then is to compute how many coupons should be drawn on average to complete the collection. This abstraction renders BioCCP fit for answering questions concerning minimal sample sizes for a wide range of combinatorial biotechnology experiments. Extrapolating to multiplex CRISPR/Cas experiments in plants, the relevant combinations of gene knockouts can be regarded as coupons, and the collector sampling from the population is the researcher performing a randomized screening experiment on a mutated plant library. For elaborate information about BioCCP, we refer to our recent paper (Van Huffel et al., 2022) and the accompanying tutorials.

##### 2.2.2.1. Plant Library Size for Full Coverage

Here, we apply BioCCP to compute E[*N*_*x,k*_] for a multiplex CRISPR/Cas experiment configured by the same experimental design settings as specified in Table 2. Figures 4, 5 demonstrate that the simulation-based results are closely approximated by the computations of BioCCP. Based on these results, we regard BioCCP as a suitable framework for gaining insight into *N*_*x,k*_ for multiplex CRISPR/Cas screens in plants. Table 3 compares the execution time to compute E[*N*_*x,k*_] for the simulation-based and BioCCP-based approaches, demonstrating a speed-up by more than two orders of magnitude when using the BioCCP-based approach. In the following, we apply other functionalities of the BioCCP package to answer additional questions related to coverage of multiplex CRISPR/Cas screens. For more information about the relevant BioCCP functions and usage, please consult Section 4.

##### 2.2.2.2. Probability of Full Coverage w.r.t. Plant Library Size

**Figure 6:**
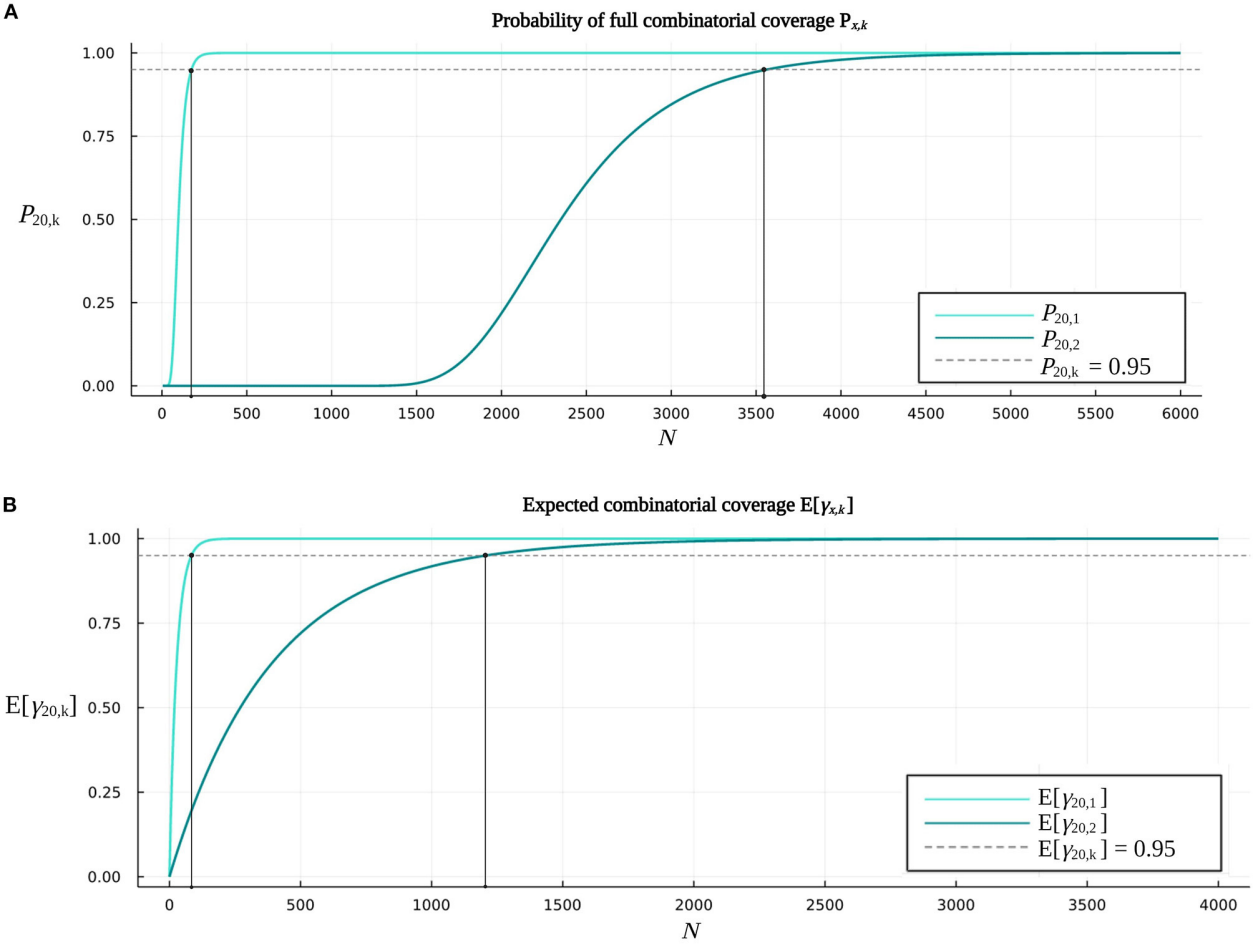
Additional functionalities provided by BioCCP to gain insight into the coverage of multiplex CRISPR/Cas screens. **(A)** The probability of full coverage *P*_20,*k*_ w.r.t. the plant library size *N* of a (multiplex) CRISPR/Cas screen. The graph *P*_20,1_ indicates the probability of full coverage of all single gene knockouts for an increasing number of plants in the plant library. Parameter settings of the CRISPR/Cas screen are specified by Table 2 (*k* = 1). The graph *P*_20,2_ quantifies the probability of full coverage of all (202) pairwise combinations of gene knockouts w.r.t. the plant library size. A multiplex CRISPR/Cas screen as specified by the parameter settings in Table 2 is considered (*k* = 2). **(B)** Expected coverage of all single gene knockouts (E[γ_20,1_]) and pairwise combinations of gene knockouts (E[γ_20,2_]) w.r.t. the plant library size of a (multiplex) CRISPR/Cas screen. Parameters of the experiments correspond to the default settings as specified in Table 2.

BioCCP provides the functionality to compute a so-called *success probability* of full coverage w.r.t. the sample size of a screening experiment. In the case of multiplex CRISPR/Cas screens, this measure indicates the probability of achieving full coverage of all *k*-combinations of gene knockouts for *x* target genes w.r.t. the number of plants analyzed in a randomized screening assay, and will be further denoted by *P*_*x,k*_. More specifically, *P*_*x*,1_ and *P*_*x*,2_, respectively, represent the probability that all single gene knockouts and all pairwise combinations of knockouts are represented at least once in a CRISPR/Cas screen with a specified plant library size *N*. Figure 6A illustrates that, for a multiplex CRISPR/Cas screen targeting double gene knockouts described by the experimental design settings in Table 2, *P*_20,2_ amounts to 0.95 when *N* is approximately equal to 3,560 plants. For an analogous screen targeting single gene knockouts, 170 plants need to be screened to obtain a probability *P*_20,1_ of 0.95. Supplementary Figure 2A visualizes the probability of full combinatorial coverage w.r.t. plant library size for a multiplex CRISPR/Cas experiment targeting triple combinations of gene knockouts.

One can also bring forward a different interpretation of *P*_*x,k*_ in the context of screens for lethal (combinations of) gene knockouts. In particular, when a specific gene knockout or combination of gene knockouts is not represented in a CRISPR/Cas screen with a plant library size corresponding to a probability *P*_*x,k*_ of 0.95, one can conclude that there is a probability of 0.95 that its absence is caused by lethality rather than non-saturation of the plant design space. Regarding the multiplex CRISPR/Cas screen targeting double gene knockouts considered in Figure 6A, one can conclude with 0.95 confidence that a pairwise combination of knockouts is not present in a mutated plant collection consisting of 3,560 randomly selected plants due to a lethal effect, and not as a result of an insufficient plant library size. By all means, this statement is only valid given that all parameter values of the multiplex CRISPR/Cas experiment used to compute *P*_*x,k*_ are properly calibrated (see Section 2.2.3).

##### 2.2.2.3. Expected Combinatorial Coverage w.r.t. Plant Library Size

We can apply BioCCP to determine the fraction of the total number of (xk) gene knockout combinations in the plant design space that is expected to be covered w.r.t. the plant library size *N* of a (multiplex) CRISPR/Cas experiment. This way, one can get insight into the expected coverage (E[γ_*x,k*_]) obtained with a given number of plants. The curve E[γ_20,2_] in Figure 6B represents the expected coverage of pairwise combinations of gene knockouts w.r.t. the plant library size of an experiment described by the parameters in Table 2. The plant library size at an expected coverage E[γ_20,2_] of 0.95 can be considered as the number of plants guaranteeing that on average 95% of all (202) pairwise combinations of knockouts will be observed at least once (Figure 6B). By way of comparison, Figure 6B also visualizes the relation between the expected value of the fraction of single gene knockouts that is observed in a CRISPR/Cas screen targeting single gene knockouts and the plant library size of the screen. Here, one can expect to cover on average 95% of all 20 single gene knockouts when including 80 plants by random selection in a plant library. For graphs visualizing the expected coverage w.r.t. plant library size for experiments targeting triple combinations of gene knockouts, please consult Supplementary Figure 2B.

#### 2.2.3. Model Calibration

In previous sections, computing *N*_*x,k*_ by means of the simulation- and BioCCP-based approaches was demonstrated for virtual CRISPR/Cas experiments. Hence, we adopted a hypothetical set of sensible parameter values in order to define the efficiency of processes at several stages of the experiment, such as the relative abundances of gRNAs in the construct library, the genome editing efficiencies of the gRNAs and the global knockout efficiency (Table 2). However, to effectively model the relation between the coverage and the plant library size of a concrete multiplex CRISPR/Cas experiment performed in the wet lab, one should calibrate the models with parameter values that approximate reality as closely as possible. For this purpose, a calibration round prior to carrying out a full-scale multiplex CRISPR/Cas experiment can be conducted. In this calibration stage, experimental data is gathered at different stages of the experiment to obtain more accurate estimates of experimental parameters.

The first type of calibration data can be generated at the level of the gRNA/Cas construct library, which is produced by vector assembly of the initial gRNA pool. At this stage, the gRNA expression cassettes in the bulk construct library can be amplified by PCR followed by next-generation sequencing, obtaining a number of reads per gRNA. As such, the relative abundances of gRNAs in the library or the empirical frequency distribution of gRNAs can be precisely determined and fed into the model. After delivery of the constructs into the target cells for the creation of the mutated plant library, a second data collection process can be executed to retrieve information about the genome editing efficiencies of the individual gRNAs. More specifically, both the gRNAs stably integrated in the genome of the mutated lines and the mutations at the associated target sites can be detected by next-generation deep sequencing (Jacobs et al., 2017; Gaillochet et al., 2021; Schaumont et al., 2022). The rate at which a specific gRNA has successfully induced a mutation at the target site determines the genome editing efficiency of the gRNA (ϵ_edit_). Lastly, the global knockout efficiency is the remaining parameter that needs to be assessed to improve the accuracy of the model. It would be of interest to develop a model that can *in silico* predict the disruption of protein function based on an observed mutated gene sequence. Such predictions could assist in identifying the fraction of mutations leading to an effective gene knockout, i.e., the empirically calibrated global knockout efficiency for a given set of gRNAs (ϵ_KO_). Schaumont et al. (2022) are currently working toward implementing a method for high-throughput multiplex gRNA design, molecular characterization of induced mutations at hundreds of loci in parallel, and automated interpretation of functional consequences of mutations, all as part of the Stack Mapping Anchor Points (SMAP) package.

After executing this calibration round, the simulation-based and BioCCP-based approaches for computing *N*_*x,k*_ can be updated by injecting the empirically measured efficiencies. As a result, one can obtain a more precise estimation of the expected minimal number of plants needed for full coverage, allowing for well-informed design of the final multiplex CRISPR/Cas experiment. For instance, it may require less effort to recalibrate a suboptimal gRNA frequency distribution in the laboratory, or to enrich the gRNA library for empirically proven active gRNAs (or to eliminate the inactive gRNAs), and repeat the construct library assembly rather than to scale up plant transformation to finally obtain a desired combinatorial coverage within a practically feasible plant library size.

### 2.3. Strategies for Increasing Coverage of Multiplex CRISPR/Cas Screens in Plants

As demonstrated by Figures 4, 5, the design parameters of a multiplex CRISPR/Cas experiment greatly impact the minimal plant library size to achieve full coverage when exploring all *k*-combinations of knockouts for *x* target genes. By means of these findings, we define two experimental design strategies that reduce the number of plants that needs to be screened for studying all relevant genetic interactions: the *Split–Select–Combine* strategy and the *Overshoot–Select–Purify* strategy.

#### 2.3.1. The *Split–Select–Combine* Strategy

**Figure 7:**
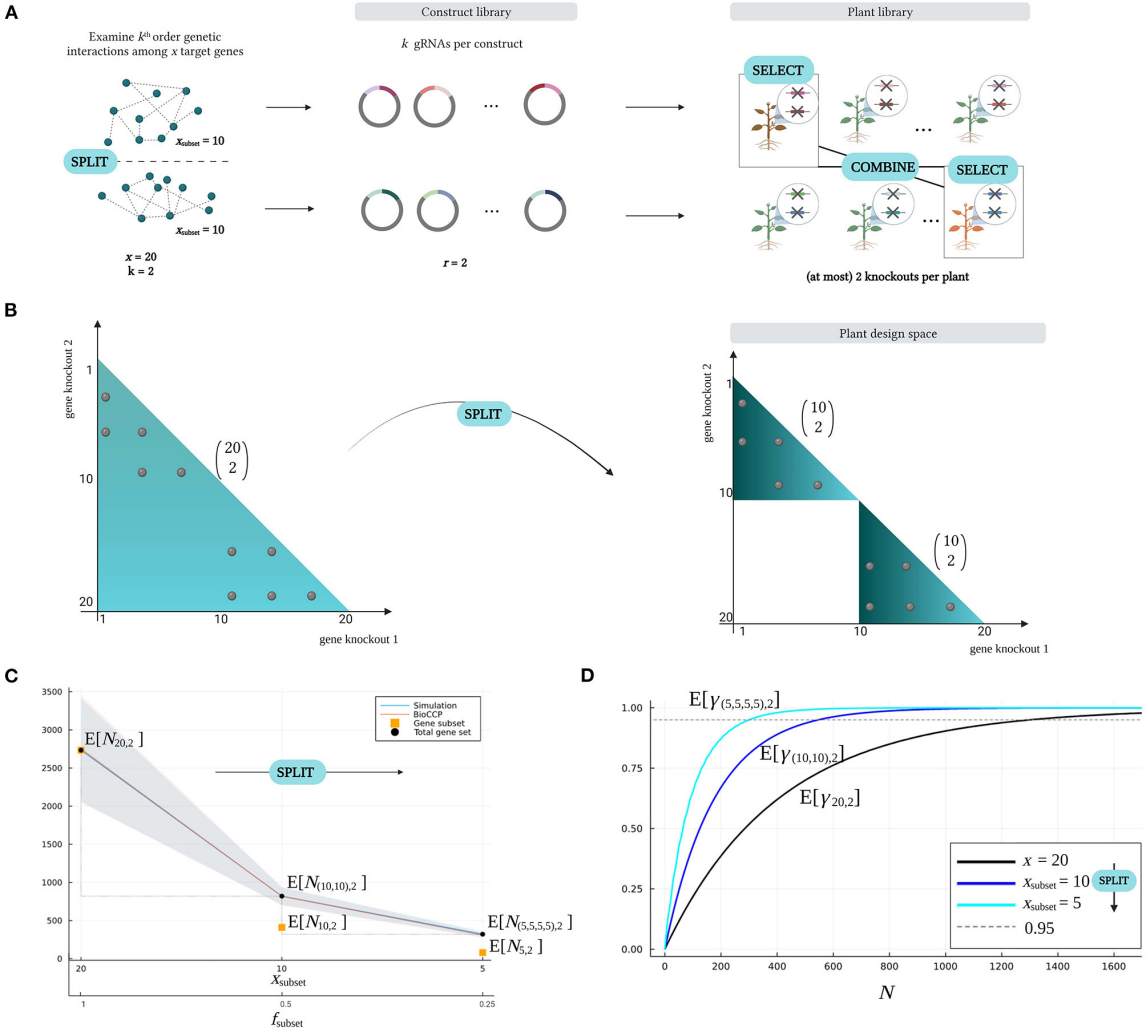
The *Split–Select–Combine* strategy. **(A)** The *Split–Select–Combine* strategy starts with dividing a set of target genes into meaningful subgroups. Here, a total number of *x* = 20 genes is divided into two subsets, each of size *x*_subset_ = 10 genes (*Split*). To study genetic interactions within these subsets separately, a construct library is created for each subset. Each construct contains two random gRNAs to explore pairwise genetic interactions (*r* = *k* = 2). Performing a phenotypic screen on the corresponding plant libraries allows to select plants with an interesting phenotype (*Select*), which can be crossed to investigate genetic interactions between the gene subsets (*Combine*). **(B)** Studying 20 target genes in two subgroups of 10 genes (*Split*) creates a plant design space that consists of two subspaces of the original design space. **(C)** Impact on the plant library size for full coverage as a total set of *x* = 20 genes is grouped into subsets of size *x*_subset_ = 10 or size *x*_subset_ = 5 (*Split*). The fraction of the total number of genes that is present in the subset is denoted by *f*_subset_. The plant library size for full coverage of all pairwise combinations of gene knockouts within the subsets separately is indicated as *N*_10, 2_ and *N*_5, 2_ (orange squares), respectively. The plant library size for full coverage of the entire gene set, is given by *N*_(10, 10), 2_ and *N*_(5, 5, 5, 5), 2_ (black dots). **(D)** Impact on the expected coverage w.r.t. plant library size as a total set of *x* = 20 genes is grouped into subsets of size *x*_subset_ = 10 or size *x*_subset_ = 5 (*Split*). Note that all parameters of the experiment (except for the number of target genes that is varied on the *x*-axis) are configured with the default settings in Table 2.

The *Split–Select–Combine* strategy studies interactions among a set of target genes in multiple distinct subsets, hence shrinking the number of possible gene knockout combinations in the plant design space and in its turn the plant library size for full coverage. Figure 7 gives a schematic overview of this approach. The *Split–Select–Combine* strategy starts with dividing a multiplex CRISPR/Cas experiment with a large number of *x* target genes into multiple screening experiments, each targeting a distinct gene subset of size *x*_subset_, addressed as the *Split* phase (Figure 7A). Grouping genes is meaningful considering that prior knowledge indicates that genes within a subset are members of the same gene family or contribute to the same metabolic or regulatory pathway, and minimally interact with genes belonging to other groups. In this regard, insights from gene family protein sequence alignments and phylogenetic analyses, genome-wide association studies, quantitative trait locus analysis and/or co-expression networks can be valuable to guide the construction of effective gene subsets (Gaillochet et al., 2021). After grouping of the genes, gRNA sequences specifically targeting each gene subset are designed. Subsequently, for each gene subset a construct library is generated, with each construct containing *k* gRNA sequences in case of exploring *k*-order genetic interactions. After transformation of these construct libraries into target cells, plant libraries exhibiting genetic perturbations in a specific gene subset are obtained. During the *Select* phase, plants with an advantageous phenotype or genotype are collected. Subsequently, further supertransformations and/or crossings between these lines can be performed to stack mutations and explore genetic interactions between different gene groups (*Combine*).

The rationale behind the *Split–Select–Combine* strategy is to search for genetic interactions in multiple, distinct design spaces with reduced combinatorial complexity (*Split*), after which valuable genotypes (*Select*) can be crossed in a more focused design space (*Combine*). Figure 7B illustrates the impact of the *Split* phase on the plant design space of a multiplex CRISPR/Cas screen. Consider a multiplex CRISPR/Cas screen targeting a total number of *x* = 20 genes for studying pairwise interactions (*k* = 2). In this case, the plant design space contains all possible (202) pairwise combinations of gene knockouts. On the contrary, the *Split–Select–Combine* strategy visualized in Figure 7B divides the target genes into two smaller subsets of size *x*_subset_ = 10 based on prior knowledge, and investigates pairwise interactions in these gene subsets separately. The plant design space now only comprises two smaller subspaces of the original design space, each containing a significantly smaller number (102) of possible gene knockout combinations. It is intuitive that this will require a smaller number of plants to achieve full saturation. As such, this strategy can contribute to optimally exploiting a limited availability of plants in multiplex CRISPR/Cas screens.

We can quantify the reduction in the plant library size for full coverage by using the *Split – Select – Combine* strategy, in particular for the default multiplex CRISPR/Cas experiment described in Table 2. Consider a total number of 20 target genes that is split into two subsets of 10 genes. The minimal number of plants for reaching full coverage γ_10, 2_ in each subset of size *x*_subset_ = 10 is denoted by *N*_10, 2_. The variable *N*_(10, 10), 2_ represents the minimal plant library size for full coverage of both subsets. For this *Split* scenario, Figure 7C demonstrates that the (102) gene knockout combinations in one gene subset can be saturated by screening on average less than E[*N*_10, 2_]≈400 plants, resulting in a total minimal plant library size of E[*N*_(10, 10), 2_]≈800 plants for entirely covering the plant design space in the *Split* scenario. When splitting the 20 target genes into four subsets of 5 genes, the minimal plant library size for full coverage of all pairwise interactions in all gene subsets even further decreases to E[*N*_(5, 5, 5, 5), 2_]≈300 plants. In contrast, a pooled screen examining all the pairwise combinations of the 20 target genes requires on average more than E[*N*_20,2_]≈2, 700 plants to saturate all (202) gene knockout combinations. Hence, the total plant library size that covers all genetic interactions in the gene subsets is substantially lower than the plant library size that saturates a single screen examining the pairwise combinations of all target genes. As previously illustrated in Figure 5A, this is due to the number of gene knockout combinations in the plant design space as well as the plant library size for full coverage increasing exponentially with a larger number of *x* target genes. Figure 7D illustrates that full coverage is reached at lower plant library size when genes are split in smaller subsets. For graphs visualizing the impact of the *Split–Select–Combine* strategy on the plant library size for full coverage and the expected coverage w.r.t. plant library size for experiments targeting triple combinations of gene knockouts, we refer to Supplementary Figure 3.

#### 2.3.2. The *Overshoot–Select–Purify* Strategy

**Figure 8:**
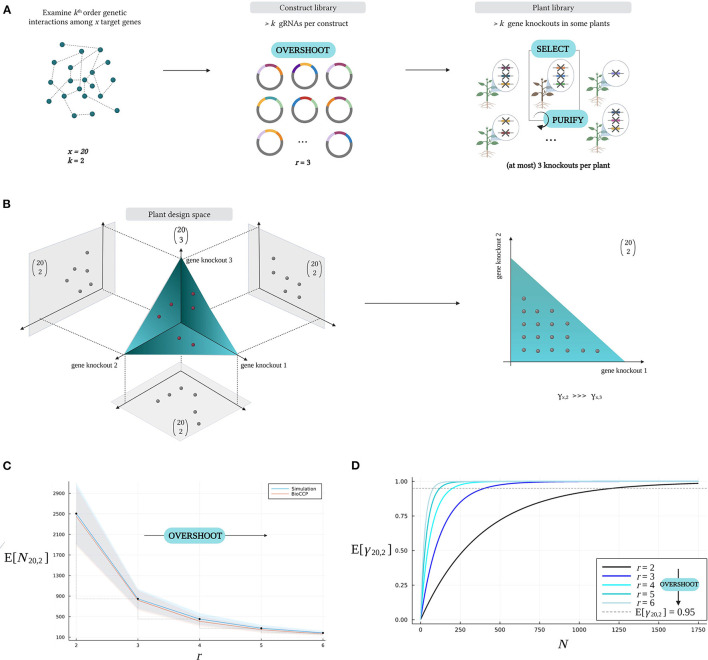
The *Overshoot–Select–Purify* strategy. **(A)** For a multiplex CRISPR/Cas experiment investigating all *k*-combinations of gene knockouts for *x* target genes, *Overshoot–Select–Purify* starts with designing a construct library with more than *k* gRNA sequences per vector (*Overshoot*). Here, pairwise interactions (*k* = 2) are investigated among *x* = 20 target genes, and *r* = 3 gRNA sequences are included per vector. The resulting plant library will contain plants with up to three gene knockouts. Interesting plant phenotypes can be collected (*Select*), after which the minimal causative genotype can be purified by Mendelian segregation (*Purify*). **(B)** Impact of *Overshoot* on the plant design space. In this example, the plant library includes six plants with each three gene knockouts, which are mapped onto the plant design space (red dots). The bag of three gene knockouts in each of the six plants can be decomposed in three pairwise combinations of gene knockouts (gray dots), resulting in a total of 18 pairwise combinations of gene knockouts. A higher coverage γ_20,2_ is reached per plant compared to a standard approach where *r* = *k* (yielding at most one pairwise combination per plant). **(C)** Reduction of *N*_*x,k*_ as a result of *Overshoot*. *N*_20,2_ decreases as a higher number of *r* gRNAs are included per construct, exploring a larger fraction of all possible pairwise combinations of gene knockouts per plant. **(D)** Increased coverage γ_20,2_ at fixed plant library size *N* as a result of *Overshoot*. In these graphs, *r* is varied, while the other parameters were configured with the default settings in Table 2.

Consider a multiplex CRISPR/Cas experiment investigating all *k*-order genetic interactions among a set of *x* target genes, hence all (xk) gene knockout combinations need to be observed at least once in the plant library. The *Overshoot–Select–Purify* strategy intends to explore more than one *k*-order genetic interaction per plant in the plant library, requiring a lower plant library size for full coverage of all (xk) gene knockout combinations. For this purpose, *Overshoot–Select–Purify* proposes to design a multiplex CRISPR/Cas experiment as illustrated in Figure 8A. After designing a collection of gRNAs specifically for the set of target genes, a construct library is generated by assembling more than *k* gRNAs per vector, which is addressed here as *Overshoot*. The generated plant library will contain plants with more gene knockouts than the order of genetic interaction under investigation (*k*). Therefore, from a geneticist's perspective, *Overshoot* implies studying multiple *k*-combinations of gene knockouts in a background of other knockouts. The latter is only valid assuming that perturbation of most target genes does not affect the phenotype of interest, rendering most background mutations neutral, and genetic interactions with an order higher than *k* are rare. For example, in Figure 8A, pairwise genetic interactions (*k* = 2) among a set of *x* = 20 target genes are investigated, and *r* = 3 gRNA sequences are assembled per gRNA/Cas construct in the construct library. The plants in the corresponding mutated library can contain up to three gene knockouts, which is considered equivalent to three specific *pairwise* combinations of gene knockouts in the *Overshoot* scenario. Upon detecting a plant with beneficial traits during the screening phase (*Select*), its genotype can be decomposed from higher-order combinations of gene knockouts through Mendelian segregation (*Purify*). In this manner, a genotype with the minimal combination of gene knockouts that is responsible for the phenotypic change can be isolated.

Figure 8B depicts the *Overshoot–Select–Purify* strategy at the level of the design space. Consider a multiplex CRISPR/Cas experiment in which up to three gene knockouts are induced per plant when investigating all pairwise genetic interactions among 20 target genes. Hence, each plant genotype in the plant library, indicated by a single point in the plant design space, is characterized by up to three gene knockouts. Here, a set of three knockouts in a plant is regarded as a “bag of three pairwise combinations” rather than a “single third-order genetic interaction.” Note that we are ignoring that each pairwise combination of gene knockouts occurs in a background of one other knockout. This illustrates that each plant with three gene knockouts allows to study up to three pairwise gene knockout combinations. In this example, six plants comprise 18 pairwise gene knockout combinations as a result of *Overshoot*. Intuitively, since a given number of plants is able to cover multiple pairwise gene knockout combinations, the plant library size for full coverage *N*_*x,k*_ is reduced.

The effect of *Overshoot* on the expected value of the plant library size for full coverage (E[*N*_*x,k*_]) is quantified in Figure 8C. As an example, consider an experiment to investigate all pairwise gene knockout combinations (*k* = 2) for a set of 20 target genes. Figure 8C visualizes the relation between the number of gRNAs per vector in the construct library (i.e., the degree of *Overshoot*) and the plant library size for full coverage (*N*_20,2_). Suppose we create a construct library with three gRNAs per vector, then the expected value of the plant library size for full coverage (E[*N*_20,2_]) is ~850 plants. In contrast, if only at most two genes per plant are knocked out, then the expected value of *N*_20,2_ amounts to more than 2,500 plants. When knocking out over three genes per plant, *N*_20,2_ decreases even more drastically (Figure 8C). Figure 8D illustrates that full coverage of all pairwise combinations of gene knockouts is reached at lower plant library size as a result of *Overshoot*. For graphs visualizing the impact of the *Overshoot–Select–Purify* strategy on the plant library size for full coverage and the expected coverage w.r.t. plant library size for experiments targeting triple combinations of gene knockouts, we refer to Supplementary Figure 4.

**Figure 9:**
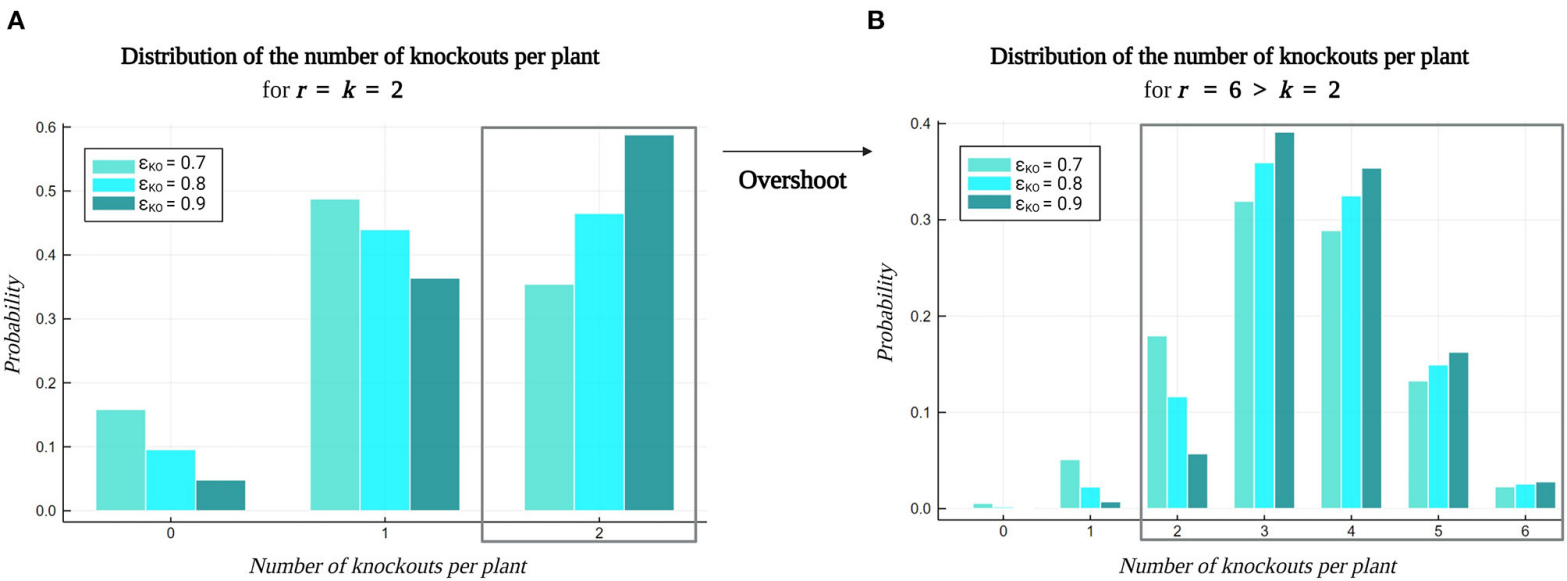
Impact of the *Overshoot–Select–Purify* strategy on the distribution of the number of knockouts per plant. A multiplex CRISPR/Cas experiment investigating pairwise combinations (*k* = 2) of gene knockouts for a set of 20 target genes was considered (see default settings in Table 2). The probability of the number of gene knockouts per plant is dependent on the global gene knockout efficiency ϵ_KO_. **(A)** The standard multiplex CRISPR/Cas approach incorporates *r* = *k* gRNAs per vector in the construct library, producing a plant library containing wildtypes, single and double knockout lines, as a result of inefficiencies related to CRISPR/Cas-mediated genome editing. **(B)** Applying *Overshoot* (*r* > *k*) yields a diverse library of plants containing relevant combinations of gene knockouts of an order that ranges from *k* to *r*.

Lastly, Figure 9 visualizes the impact of *Overshoot* on the distribution of the number of knockouts per plant in a library. Multiplex CRISPR/Cas experiments targeting double gene knockouts (*k* = 2) with varying global knockout efficiency ϵ_KO_ are considered. Figure 9A depicts the distribution of the number of knockouts per plant resulting from multiplex CRISPR/Cas experiments employing a standard approach, involving the generation of a construct library with the number of gRNAs per vector equal to the order of genetic interaction to investigate (*r* = *k* = 2). However, due to inefficiencies during CRISPR/Cas-mediated genome editing, a large fraction of the plants in the resulting plant library will carry a knockout in only one target gene, or might not possess a gene knockout at all. As these lower-order mutated lines do not enable the study of any pairwise combinations of gene knockouts, they do not contribute to covering relevant *k*-combinations of gene knockouts in the combinatorial design space. Figure 9A illustrates that these plants occupy a larger part of the plant library as the global knockout efficiency ϵ_KO_ decreases. On the contrary, in Figure 9B, an *Overshoot* scenario is depicted, in which six gRNAs are included per gRNA/Cas construct (*r* = 6), while targeting pairwise combinations of gene knockouts (*k* = 2). Here, all plants in the library that hold a number of gene knockouts that is equal to or greater than *k*, i.e., all double to sextuple knockout lines, are valuable for covering *k*-combinations of knockouts (possibly in a background of other knockouts, in this case providing multiple *k*-combinations per plant). This way, on the one hand, *Overshoot* is able to optimally exploit a set of plants for combinatorial coverage, compensating for inefficiencies during CRISPR/Cas-mediated gene perturbation. On the other hand, note that *Overshoot* is also able to leverage these inefficiencies as a tool to create diverse orders of combinations of gene knockouts in a single plant library.

Importantly, a large fraction of the mutated lines resulting from *Overshoot* contains combinations of gene knockouts of an order higher than the order of genetic interaction under investigation. These higher-order combinations potentially hold mutations additional to the minimal set of gene knockouts that is causative for the phenotype of interest. Therefore, after the selection of individuals with relevant phenotypes from the mutated collection (*Select*), the *Purify* step involves the isolation of the effective combinations of gene knockouts through several backcrosses. Backcrossing implies subjecting lines to crossings with plants from a different genetic background in order to achieve a progeny in which the higher-order combinations of gene knockouts segregate into lower-order combinations. Then, individuals with interesting phenotypes are once again selected from the progeny, whereafter genotyping through massively parallel sequencing serves to identify the set of mutations that is associated with the observed phenotype. An iterative process of estimating genotype-phenotype associations and Mendelian segregation through several backcrosses may be necessary to arrive at the minimal causative set of gene knockouts. The total number of plants that needs to be generated, phenotyped, and genotyped during the *Purify* phase depends on the order of genetic interaction that is responsible for the desired phenotype. In fact, isolating a lower-order combination of gene knockouts from a higher-order mutational background implies substantially shrinking the initial search space, requiring an increased number of backcrosses. Therefore, the trade-off between the advantage of minimizing the scale of the initial CRISPR/Cas screen by *Overshoot* on the one hand, and the substantial amount of resources and efforts for purifying the genotypes afterwards on the other hand, is decisive for the ideal number of knockouts per plant. Note that the quantification of the number of plants that needs to be generated, phenotyped, and genotyped during *Purify* is left for future work. In addition, it should be emphasized that an increasing number of gRNAs per gRNA/Cas construct (more than six gRNAs per gRNA/Cas construct) may result in competition among the individual gRNAs for the common Cas nuclease core and hence reduced genome editing efficiencies (Stuttmann et al., 2021). This reduced efficiency imposes additional constraints on the degree of *Overshoot* that can be applied in a multiplex CRISPR/Cas experiment.

## 3. Discussion

The development of multiplex CRISPR/Cas systems has advanced the study of genetic interplay in biological processes, by allowing for the targeted mutation of multiple genes simultaneously in a single cell or plant line. This technology has the potential to improve understanding about how synergistic, additive, and/or redundant gene function impacts complex agronomic traits in various plant species, and hence facilitate the development of optimal plant phenotypes. In this study, we focused on the application of the multiplex CRISPR/Cas system in plants to investigate all *k*-order genetic interactions among *x* target genes. To that end, it is of primary importance that the plant library contains all possible *k*-combinations of gene knockouts in the plant design space. Otherwise, one cannot distinguish whether a particular gene knockout combination is missing in the set of plants with a relevant phenotype due to an insufficient plant library size or whether a particular combination of gene knockouts is lethal. Moreover, when only a fraction of all relevant combinations of gene knockouts is represented in the multiplex CRISPR/Cas screen, effective genetic interactions and complex genotype-phenotype associations are discovered merely by chance. To avoid misleading conclusions, the design of such experiments must correct for full coverage of all (xk) combinations of gene knockouts in the design space. For this purpose, a sufficient number of plants needs to be included in the plant library by random sampling. Notably, current protocols for multiplex CRISPR/Cas screens in plants lack guidelines in this regard. In this study, the *plant library size* of a multiplex CRISPR/Cas experiment that achieves full coverage was referred to as *N*_*x,k*_. The central objective of this study was to develop tools for quantifying the expected value and standard deviation of *N*_*x,k*_. Note that we focus on a minimal plant library size guaranteeing the representation of all relevant knockout combinations at least once in the plant library. However, the latter forms a theoretical lower bound to the actual number of plants that is required for a researcher to effectively link gene knockout combinations to qualitative or quantitative phenotypic effects. In practice, an even larger plant library size will have to be considered to distinguish true effects from phenotypic noise resulting from biological variation and false positive results due to off-target CRISPR/Cas editing activity or spontaneous mutations. In particular, to ensure a minimal level of statistical power, the plant library size for the reliable detection of effects will increase with a higher phenotypic variation among biological replicates.

To contribute to well-informed experimental design of multiplex CRISPR/Cas screens in plants, we provided two approaches to gain insights into the plant library size guaranteeing full coverage of all *k*-combinations of gene knockouts for *x* target genes. First, a simulation-based approach was presented, which repeatedly generates mutated plant lines *in silico* until all (xk) gene knockout combinations in the plant design space are fully covered. These simulations reflect subsequent stages throughout a multiplex CRISPR/Cas experiment, modeling several sampling processes starting from the assembly of gRNA/Cas constructs to the CRISPR/Cas-mediated genome editing of plant cells and the collection of a library of mutated plants. In order to closely resemble a realistic scenario, imbalances in the relative abundance of gRNAs and inefficiencies on the level of genome editing and the induction of loss-of-function mutations were taken into account. These parameters can be varied to assess their impact on the expected value and the standard deviation of the plant library size for full coverage. An advantage of the simulation-based approach is its transparency, providing a clear overview of the subsequent stages and associated efficiencies of a multiplex CRISPR/Cas experiment. As a consequence, information regarding the separate stages can be extracted for deeper understanding of, e.g., the composition of the construct library, the distribution of mutations after genome editing and the distribution of gene knockouts in the plant library for a specified set of experimental design parameters. Alternatively, we provided the comprehensive BioCCP-based approach, translating the computation of an adequate plant library size for multiplex CRISPR experiments into a variant of the Coupon Collector Problem (CCP). This higher level of abstraction allows for a quick estimate of the expected value and standard deviation of the minimal number of plants needed for full coverage, yielding immediate insights into the practical feasibility and potential cost and effort of genotyping and phenotyping of a specific multiplex CRISPR/Cas experiment. Both the simulation- and BioCCP-based approach can be easily repurposed for multiplex CRISPR/Cas experiments based on other gRNA delivery methods than *Agrobacterium* transformation of multiplex gRNA/Cas constructs, such as protoplast transfection or particle bombardment (Cunningham et al., 2018; Liang et al., 2018; Toda et al., 2019). Furthermore, they may be extended to account for additional processes and efficiencies associated with a CRISPR/Cas experiment (e.g., vector propagation in a bacterial host, plant cell transformation, and plant regeneration). It should be noted that the BioCCP-based approach offers an approximate solution, as a result of abstracting combinations of gene knockouts into independent modules and neglecting their dependence when multiple gene knockouts are present per plant. The simulation-based approach will deliver an accurate solution, given that the efficiency parameters are well-calibrated and that a sufficient number of repetitions is performed during the simulation. Notwithstanding, the BioCCP-based approach offers a more computationally friendly way for computing *N*_*x,k*_, demonstrating speed improvements of more than two orders of magnitude. Therefore, the BioCCP package was employed to develop complementary tools for studying the coverage γ_*x,k*_ of a plant library. As such, the BioCCP-based approach allows to compute the probability of full coverage *P*_*x,k*_ w.r.t. the plant library size *N* for a specific experiment. Further, insights regarding the expected coverage E[γ_*x,k*_] w.r.t. a given plant library size can be gained. This precise quantification of the representation of gene knockout combinations in function of plant library size has not yet been systematically addressed in published studies. Both measures facilitate a deeper understanding of adequate plant library sizes for multiplex CRISPR/Cas experiments.

Moreover, we illustrated the impact of several experimental design parameters on the expected value of *N*_*x,k*_, improving understanding of how adjustments of design settings can contribute to minimizing the number of plants that should result from a multiplex CRISPR/Cas experiment to achieve full coverage. Importantly, an increasing number of target genes results in an explosion of the combinatorial plant design space and hence also brings about a rapid increase in the associated plant library size guaranteeing its full coverage (Figure 5). Equally important is the order of genetic interaction (*k*) one intends to study, since a combinatorial explosion of the design space occurs as higher-order interactions are to be investigated (Table 3). The latter clearly indicates that the number of target genes and order of genetic interaction investigated in an experiment when imposing full coverage is strongly constrained by the manageable number of plants for genotyping and phenotyping. Hence, designing a CRISPR/Cas experiment in a naive way might lead to an unfeasible plant library size for covering the combinatorial design space. Most current studies on multiplex CRISPR/experiments investigate genetic interactions among a relatively small pool of 5 to 15 target genes (Ma et al., 2015; Zhang et al., 2016; Jacobs et al., 2017; Shen L. et al., 2017; Miao et al., 2018; Li et al., 2019; Bai et al., 2020; Lin et al., 2020; Rojas-Murcia et al., 2020; Trogu et al., 2021), or intend to assess the individual phenotypic effects of a large number of 50–13,000 genes (Meng et al., 2017; Liu H.J. et al., 2020; Zhang N. et al., 2020; Chen et al., 2022). In these papers, a thorough assessment regarding the coverage of (combinations of) gene knockouts in the CRISPR/Cas screen to determine an adequate plant library size is lacking. We highlight that although the emergence of efficient multiplex CRISPR/Cas systems is rendering the generation of high-order mutant plant libraries technologically realizable (Shen L. et al., 2017; Miao et al., 2018; Stuttmann et al., 2021; Trogu et al., 2021), typical plant library sizes only allow for a limited number of gene knockout combinations to be properly investigated. Additionally, inefficiencies at different stages of the multiplex CRISPR/Cas protocol play a major role, as *N*_*x,k*_ increases significantly with: (1) an unequal abundance distribution of gRNA sequences in the gRNA/Cas construct library, (2) inferior genome editing efficiencies of gRNAs, and (3) a lower fraction of mutations leading to loss-of-function of the gene product, i.e., a reduced global knockout efficiency. These factors form potential bottlenecks in achieving full coverage (γ_*x,k*_ = 1) in multiplex CRISPR/Cas screens. It was shown that the effect of unequal abundances and inferior genome editing efficiencies of gRNAs on *N*_*x,k*_ can be mitigated by designing more gRNAs per target gene. For the sake of future work in the multiplex CRISPR/Cas field, the quantitative analysis presented in this paper raises awareness about the limitations on the order of genetic interaction that can be investigated among a number of target genes, given that the feasible number of plants in a screening assay is confined and that inefficiencies at several stages of the multiplex CRISPR/Cas protocol are inevitable. Our model provides the opportunity to calibrate all stages of the experiment “on the fly” by injecting the empirically observed gRNA abundances and efficiency distributions into the model and updating all estimates accordingly. It should be acknowledged that there exist additional inefficiencies and bottlenecks in the multiplex CRISPR/Cas protocol that are not included in our models, which might further magnify *N*_*x,k*_. For example, if heterozygous and homozygous mutations are considered as separate genotypic states, the combinatorial design space that is to be covered inflates even further.

An additional goal of this work was to suggest experimental design strategies to construct multiplex CRISPR/Cas screens in plants with a lower *N*_*x,k*_. First, we proposed the *Split–Select–Combine* strategy, which groups target genes into meaningful subsets based on prior knowledge, resulting in multiple screens with a lower total combinatorial complexity compared to a single screen encompassing all target genes. As a result, full coverage can be reached at a reduced *N*_*x,k*_. Second, the *Overshoot–Select–Purify* strategy was presented. Here, the number of knockouts induced per plant is larger than the order of genetic interaction under investigation (*k*) in order to study multiple *k*-order genetic interactions per plant. In this manner, one intends to initially span as much as possible relevant combinations of gene knockouts with a limited number of plants. Afterwards, relevant areas in the combinatorial design space can be more thoroughly explored by Mendelian segregation and the minimal causative genotype can be purified. Furthermore, it should be highlighted that, without altering the experimental design strategy, coverage of a multiplex CRISPR/Cas screen can be enhanced by minimizing inefficiencies at several stages of the experiment. For instance, as seen from Figure 5C, a more equal distribution of the relative frequencies of the gRNAs in the construct library might substantially lower *N*_*x,k*_. Hence, there is a need for approaches to mitigate biases during the synthesis, quantification, and cloning of gRNA sequences that result in specific gRNAs to be over- or underrepresented in a construct library (Wegner et al., 2019; Imkeller et al., 2020). Further, *N*_*x,k*_ can be reduced by optimizing the genome editing activities of gRNAs. The latter implies improvements in gRNA design to maximize the genome editing rate and minimize off-target activity, diminishing the occurrence of false negatives and false positives, respectively. Various web-based tools for gRNA design have been developed (Gerashchenkov et al., 2020). Furthermore, the gRNA pool may be enriched for biologically active gRNAs after initial *in vitro* or *in planta* high-throughput screens, and inactive gRNAs may be removed before construct library assembly. In addition, testing multiple nuclease orthologs of the Cas protein to identify highly efficient variants and the use of alternative promotors driving the expression of the Cas nuclease can boost genome editing efficiency (Bortesi et al., 2016; Najm et al., 2018; Hassan et al., 2021). Moreover, it is critical that the gRNAs are designed to specifically target functional protein domains and that the editing outcome results in loss-of-function of the protein, improving the global knockout efficiency. We anticipate that implementing these guidelines will greatly increase effective coverage of relevant gene knockout combinations in multiplex CRISPR/Cas screens in plants as well as contribute to the correct interpretation of these screening experiments.

## 4. Materials and Methods

All code accompanying the simulation- and BioCCP-based approaches was run in Julia-Jupyter Notebook and is available at https://github.com/kirstvh/MultiplexCrisprDOE. Runtime experiments were performed on an Intel core i7 2.60 GHz processor machine with 32 Gbytes of RAM and a 64-bit operating system.

### 4.1. Simulation-Based Approach

#### 4.1.1. gRNA Relative Frequency Distribution and *in silico* Vector Assembly

During the simulation procedure, each gRNA in the library is assigned a read number by random sampling from the gRNA frequency distribution. This distribution is characterized by a fixed ratio ρ of the frequency of the most abundant gRNA to the frequency of the least abundant gRNA. More specifically, the gRNA frequency distribution is defined as a double truncated normal distribution, with a lower bound of truncation (*l*) and upper bound of truncation (*u*) such that $\rho =\frac{l}{u}$. Note that only the ratio $\frac{l}{u}$, and not the exact value of *l* and *u*, is crucial, since downstream in the simulation a normalization step is performed on the gRNA reads in order to obtain a series of probabilities that add up to 1 (determining the sampling probability of each gRNA to be included in a vector of the construct library). The expectation of the normal distribution (μ) is set to $\frac{l+u}{2}$ and the standard deviation (σ) is set to $\frac{u-l}{2}$. In the default scenario, the following settings are applied: *l* = 50, *u* = 100, μ = 75, σ = 25, resulting in ρ = 2. The histogram of the gRNA abundances is depicted in Figure 3A. Relative frequencies are calculated by normalizing the abundances to add up to 1. Each gRNA/Cas construct is assembled *in silico* by sampling *k* gRNAs according to a multinomial distribution with the probability of sampling each gRNA being equal to its relative frequency in the gRNA library.

#### 4.1.2. gRNA Genome Editing Efficiency Distribution, Global Knockout Efficiency, and *in silico* Genome Editing

After generating a gRNA/Cas construct *in silico*, this vector is assumed to be transformed and consequently expressed in a target cell with 100% efficiency. The genome editing process is simulated as follows. A genome editing efficiency is attributed to each gRNA. By default, *f*_act_ = 90% of the gRNAs is assumed to be highly active. For these active gRNAs, the genome editing efficiency is obtained by sampling from a normal distribution with μ = 0.95 (denoted as ϵ_edit,act_) and σ = 0.01. The remaining (1 − *f*_act_) = 10% of all gRNAs is assigned a low activity, drawing a genome editing efficiency from a normal distribution with μ = 0.1 (denoted as ϵ_edit,inact_) and σ = 0.01. This sampling procedure is equivalent to drawing genome editing efficiencies from a bimodal distribution (see also the CRISPR/Cas simulation study of Nagy and Kampmann, 2017). To model the induction of mutations in the target cell, for each gRNA present in the construct a value is drawn from a Bernoulli distribution with *p* equal to the genome editing efficiency ϵ_edit_ of the gRNA. This sample takes the value 1 with probability *p* = ϵ_edit_, simulating the effective induction of a mutation, and takes the value 0 with probability 1 − ϵ_edit_, representing the absence of a mutation. After deciding on whether a gRNA has effectively induced a mutation, another Bernoulli distribution is used to model whether a mutation results in a loss-of-function gene knockout. Here, the Bernoulli parameter *p* is equal to global gene knockout efficiency ϵ_KO_, which describes the fraction of gene edits resulting in a loss-of-function mutation and which is equal for all gRNAs. By default, ϵ_KO_ is set at a value of 0.8. The foregoing sampling process is executed for each gRNA of a virtually transformed construct in order to determine whether the gRNA has effectively knocked out the target gene in the plant cell. At the end, the corresponding plant is characterized by a specific set of gene knockouts.

#### 4.1.3. Computation of the Expected Value and the Standard Deviation of the Plant Library Size for Full Coverage

Plants are virtually collected and the observed combinations of gene knockouts are stored. The repeated sampling of plant genotypes ends when all genetic interactions of interest have been targeted, i.e., full coverage of all (xk) gene knockout combinations in the combinatorial design space is achieved. The plant library size at which this goal is realized, is stored as the *N*_*x,k*_ of the current trial of the multiplex CRISPR/Cas experiment. To obtain an expected value and standard deviation of the plant library size for full coverage, each specific multiplex CRISPR/Cas experiment is simulated 500 times.

Note that the relative abundances in the construct library and genome editing efficiencies of the gRNAs are randomly sampled from a distribution. The outcome of this stochastic process is dependent on the seed value that is used to initialize the pseudo-random number generator. Therefore, the series of relative abundances and genome editing efficiencies of the gRNAs, and hence the value of E[*N*_*x,k*_] and σ[*N*_*x,k*_], will vary with the chosen seed. Therefore, when investigating the influence of ρ of the gRNA frequency distribution and *f*_act_ of the genome editing efficiency distribution on E[*N*_*x,k*_] and σ[*N*_*x,k*_] in Figures 4, 5C,D, E[*N*_*x,k*_] and σ[*N*_*x,k*_] are computed for several series of gRNA frequencies and genome editing efficiencies corresponding to a specific ρ and *f*_act_, respectively. The different outcomes for E[*N*_*x,k*_] and σ[*N*_*x,k*_] are averaged.

### 4.2. BioCCP-Based Approach

BioCCP is a general framework focusing on determining sample sizes for screening experiments in combinatorial biotechnology that guarantee full coverage of the design space (Van Huffel et al., 2022). BioCCP requires the input of the total number of distinct modules in a design space, the number of modules per design and the probability distribution of the modules, describing the probability of being included in a design, in order to define the design space and compute its statistical properties (e.g., how many designs should be sampled on average to observe each module at least once, or what the expected coverage of all modules is w.r.t. a given sample size). In the following, we describe the translation of a CRISPR/Cas experiment into the BioCCP framework.

#### 4.2.1. Definition of Inputs

The problem setting of calculating the expected value of the minimal plant library size for full coverage of all single gene knockouts (E[*N*_*x*,1_]) in a CRISPR/Cas screen is translated into BioCCP terms as follows. Each plant design is regarded as an assembly of single gene knockouts, and the specific goal is to collect a set of plants that spans each possible gene knockout at least once. Therefore, the number of possible single gene knockouts (which is equal to the number of target genes) is fed into the BioCCP model as the number of distinct modules in the design space that needs to observed at least once. For an experiment targeting single gene knockouts, by default one gRNA is included per gRNA/Cas construct. Accordingly, the number of modules per design is set to the value of 1. The probability to encounter a knockout in a gene (module) is calculated by summing up the probabilities of the relevant gRNAs (the gRNAs specifically designed to target this gene) to induce a knockout in this gene, taking into account the relative frequencies of the relevant gRNAs in the construct library, the genome editing efficiencies of the gRNAs and the global gene knockout efficiency. The relative frequencies and genome editing efficiencies of the gRNAs are sampled according to the distributions described in Sections 4.1.1 and 4.1.2.

For examining the expected plant library size for full coverage of all *k*-combinations of gene knockouts (E[*N*_*x,k*_]) in multiplex CRISPR/Cas screens (*k*>1), abstraction into BioCCP terms implies the following. First, the number of *k*-combinations of gene knockouts is considered as the total number of modules to be collected. Secondly, the number of modules per design is set as the number of gRNA combinations per vector in the gRNA/Cas construct library. For instance, a construct library with six gRNA sequences per vector contains 15 pairwise combinations, corresponding to 15 modules per design. The probability to encounter a gene knockout combination (module) in a plant (design) is computed as follows. For each gene knockout combination, the corresponding combinations of gRNAs are listed. The probability of encountering a combination of gRNAs in the construct library is calculated by multiplying the relative frequency of the individual gRNAs in the construct library and subsequent normalization of all probabilities to add up to one. Thereafter, the genome editing efficiency for each gRNA in the combination and the global knockout efficiency are incorporated to obtain the probability of all gRNA combinations to induce an effective combination of gene knockouts. Finally, the probability of encountering a specific *k*-combination of gene knockouts is obtained by summing up the probabilities of all corresponding gRNA combinations to induce this particular *k*-combination of gene knockouts in a plant.

#### 4.2.2. Computation of the Expected Value and Standard Deviation of the Plant Library Size for Full Coverage and Other Relevant Statistics

After definition of the above-mentioned inputs, the BioCCP functions are employed to compute statistics related to the plant library size for full coverage (*N*_*x,k*_). The functions BioCCP.expectation_minsamplesize and BioCCP.std_minsamplesize are used to compute respectively E[*N*_*x,k*_] and σ[*N*_*x,k*_] of a given multiplex CRISPR/Cas experiment. Computation of the expected coverage (E[γ_*x,k*_]) w.r.t. the plant library size is carried out by applying the BioCCP.expectation_fraction_collected function. Finally, the computation of the probability of full coverage (*P*_*x,k*_) involves employing the function BioCCP.success_probability.

Documentation describing the precise computation of these statistics is available at https://github.com/kirstvh/MultiplexCrisprDOE. This GitHub repository also provides customized functions for automatically converting the characteristics of a multiplex CRISPR/Cas experiment into BioCCP terms/inputs.

## Data Availability Statement

The data and source code generated for this study can be found in a GitHub repository (https://github.com/kirstvh/MultiplexCrisprDOE) under an MIT software license. A Galaxy version of the tool is available to be installed at the Galaxy ToolShed and to be used directly at usegalaxy.be.

## Author Contributions

KV, MS, TR, and BD conceptualized the study. KV wrote the source code for the computational frameworks and performed the simulation experiments. All authors contributed to writing the original draft of the manuscript and approved the submitted version.

## Funding

This research received funding from the Flemish Government under the Onderzoeksprogramma Artificiële Intelligentie (AI) Vlaanderen programme. KV holds a doctoral mandate [Grant number BOF21/DOC/154] of the Bijzonder Onderzoeksfonds (BOF).

## Conflict of Interest

The authors declare that the research was conducted in the absence of any commercial or financial relationships that could be construed as a potential conflict of interest.

## Publisher's Note

All claims expressed in this article are solely those of the authors and do not necessarily represent those of their affiliated organizations, or those of the publisher, the editors and the reviewers. Any product that may be evaluated in this article, or claim that may be made by its manufacturer, is not guaranteed or endorsed by the publisher.
