# Supplementary material for: Covering the Combinatorial Design Space of Multiplex CRISPR/Cas Experiments in Plants
Source: Front Plant Sci. 2022 Jun 20;13:907095. doi: 10.3389/fpls.2022.907095 (PMC9251496; doi:10.3389/fpls.2022.907095)
Supplement: Supplementary file 1 [file Data_Sheet_1.pdf]

Supplementary Material

1 SUPPLEMENTARY DATA

The supplementary Figures in this section consider multiplex CRISPR/Cas experiments targeting triple combinations of gene knockouts (as specified by the default settings in Table 2 in the manuscript; $k = 3$). The impact of design parameters on the plant library size for full coverage of all triple combinations is illustrated in Figure S1. The probability of full coverage and the expected combinatorial coverage w.r.t. a given plant library size are visualized in Figure S2. In Figure S3,S4, the impact of the *Split–Select–Combine* strategy and *Overshoot-Select–Purify* strategy on the the plant library size for full coverage and the expected coverage w.r.t. plant library size is demonstrated. Code to reproduce these Figures can be found at `https://github.com/kirstvh/MultiplexCrisprDOE`.

1.1 Supplementary Figures

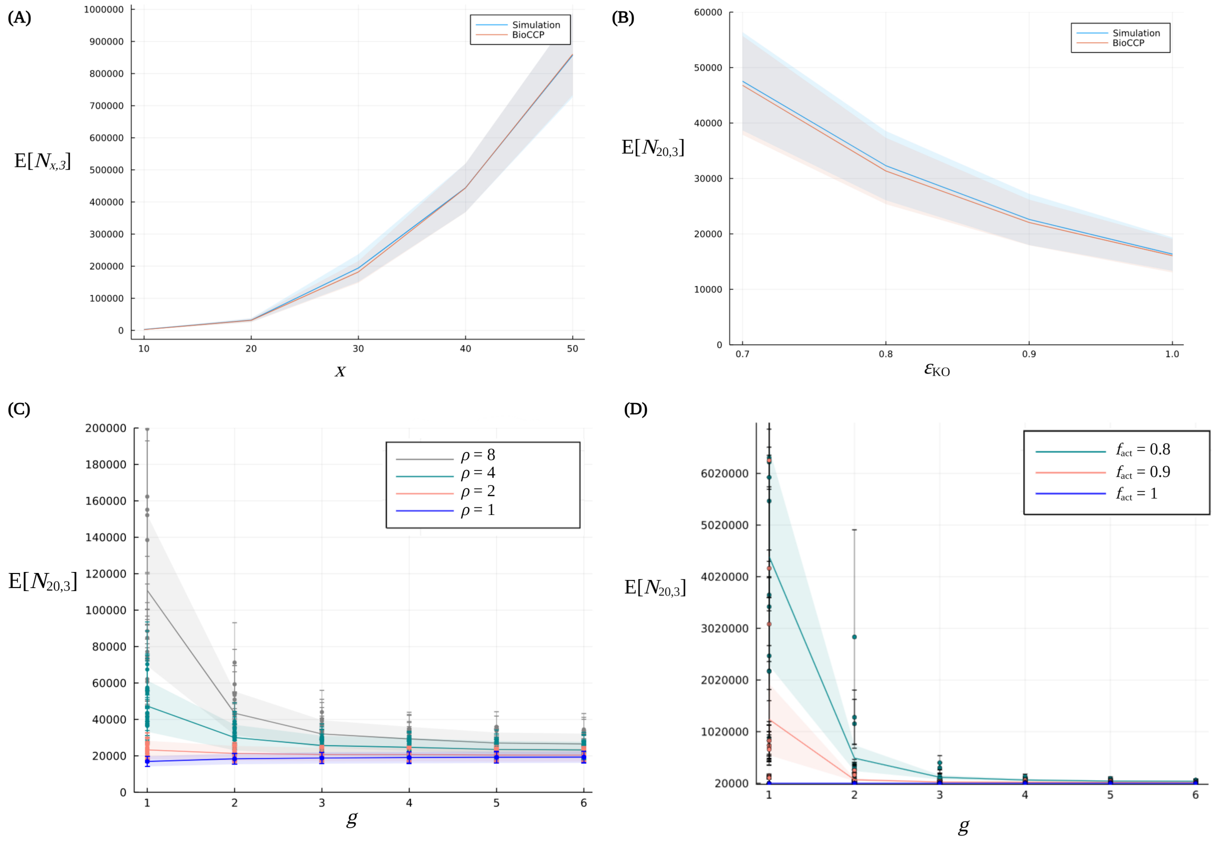

Figure S1. Relation between the expected value of the plant library size for full coverage of triple combinations of gene knockouts and design parameters of a multiplex CRISPR/Cas experiment. **(A)** Effect of an increasing number of x target genes on $\mathrm{E}[N_{x,3}]$. **(B)** Effect of the global knockout efficiency ϵ_{KO} on $\mathrm{E}[N_{20,3}]$. The blue curve indicates the simulation-based results, while the red curve indicates the results of the BioCCP framework. The width of the shaded area around these curves represents $\sigma[N_{20,3}]$. In each of the graphs, the parameter under investigation is varied while the values of the other parameters are fixed at the default settings as specified in Table 2 in the manuscript ($k = 3$). **(C)** Combined effect of parameter ρ of the gRNA frequency distribution for an increasing number of g gRNAs per gene on $\mathrm{E}[N_{20,3}]$. **(D)** Effect of the fraction f_{act} of active gRNAs for an increasing number of g gRNAs per gene on $\mathrm{E}[N_{20,3}]$.

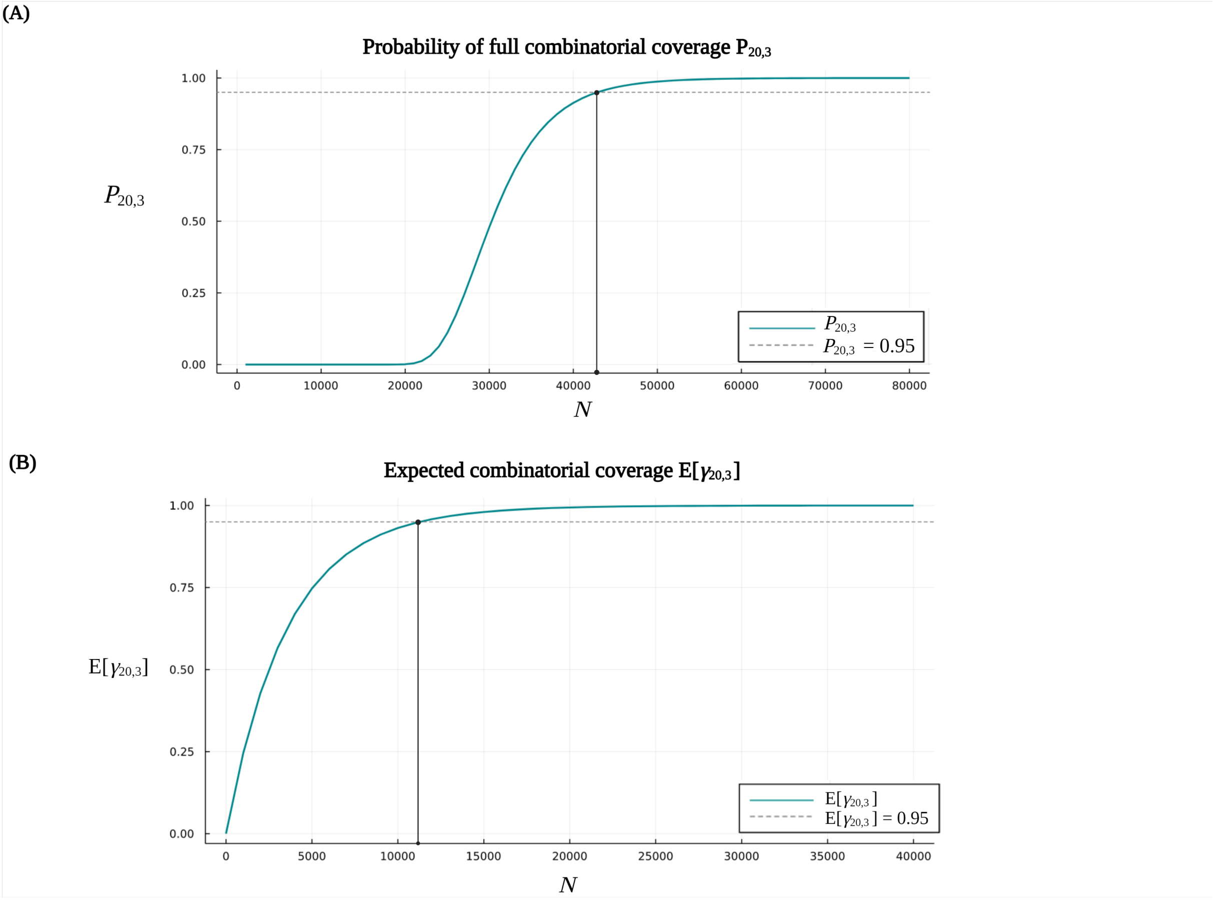

Figure S2. Additional functionalities provided by BioCCP to gain insight into the combinatorial coverage of multiplex CRISPR/Cas screens. Consider an experiment targeting triple combinations of gene knockouts ($k = 3$), specified by the parameter settings in Table 2 in the manuscript. **(A)** The probability of full coverage of all triple combinations of gene knockouts ($P_{20,3}$) w.r.t. the plant library size N. **(B)** Expected coverage of all triple combinations of gene knockouts ($\mathrm{E}[\gamma_{20,3}]$) w.r.t. the plant library size.

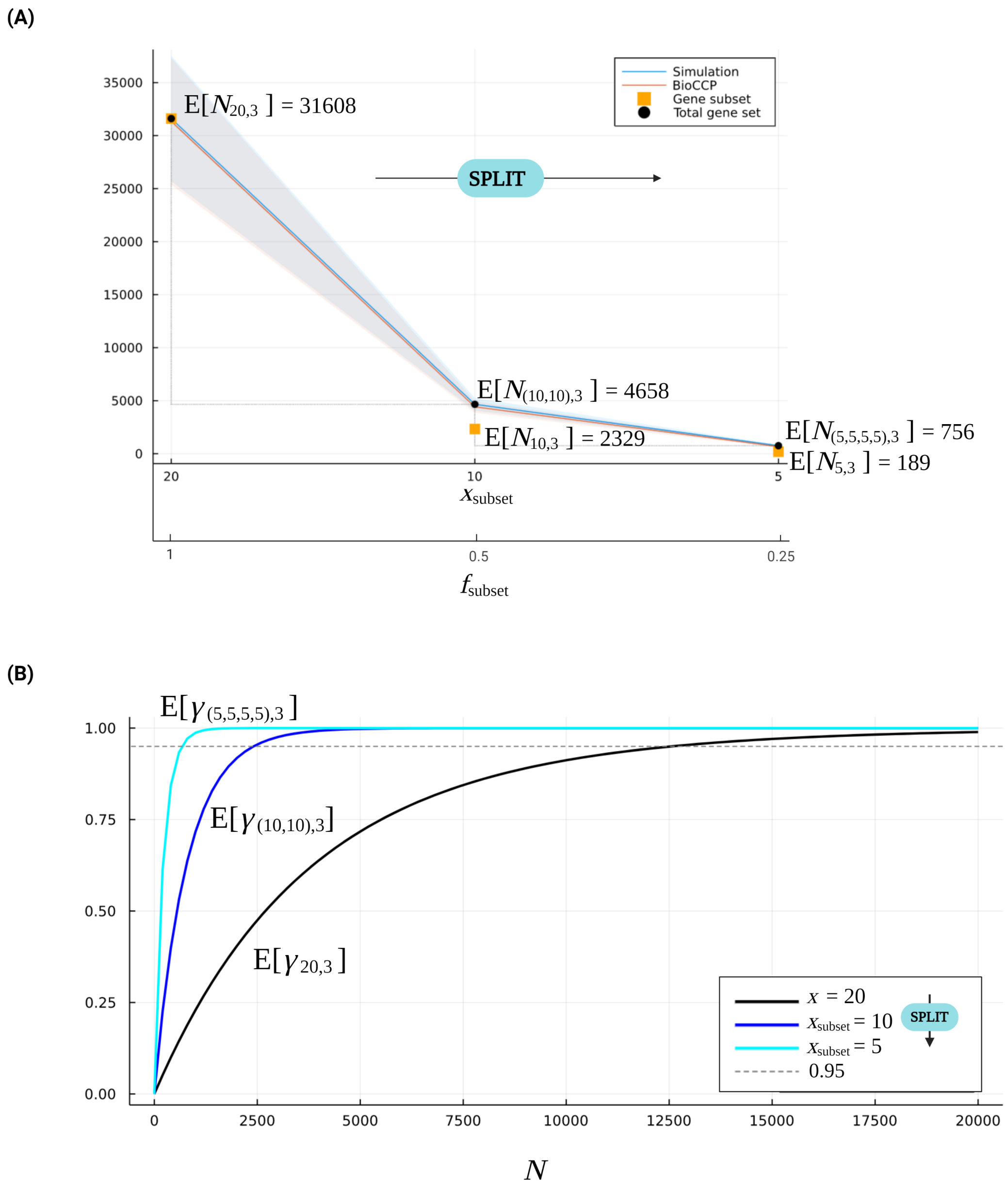

Figure S3. The *Split–Select–Combine* strategy. Consider a multiplex CRISPR/Cas experiment targeting triple combinations of gene knockouts, as specified by the default settings in Table 2 in the manuscript ($k = 3$). **(A)** Impact on the plant library size for full coverage of all triple combinations of gene knockouts as a total set of $x = 20$ genes is grouped into subsets of size $x_{\text{subset}} = 10$ or size $x_{\text{subset}} = 5$ (*Split*). The fraction of the total number of genes that is present in the subset is denoted by f_{subset}. The plant library size for full coverage of all triple combinations of gene knockouts within the subsets separately is indicated as $N_{10,3}$ and $N_{5,3}$ (orange squares), respectively. To obtain the plant library size for full coverage of the entire gene set (black dots), the plant library sizes to saturate all gene knockout combinations in the gene subsets are summed for all subsets, resulting in $N_{(10,10),3}$ and $N_{(5,5,5,5),3}$, respectively. **(B)** Impact on the expected combinatorial coverage w.r.t. plant library size as a total set of $x = 20$ genes is grouped into subsets of size $x_{\text{subset}} = 10$ or size $x_{\text{subset}} = 5$.

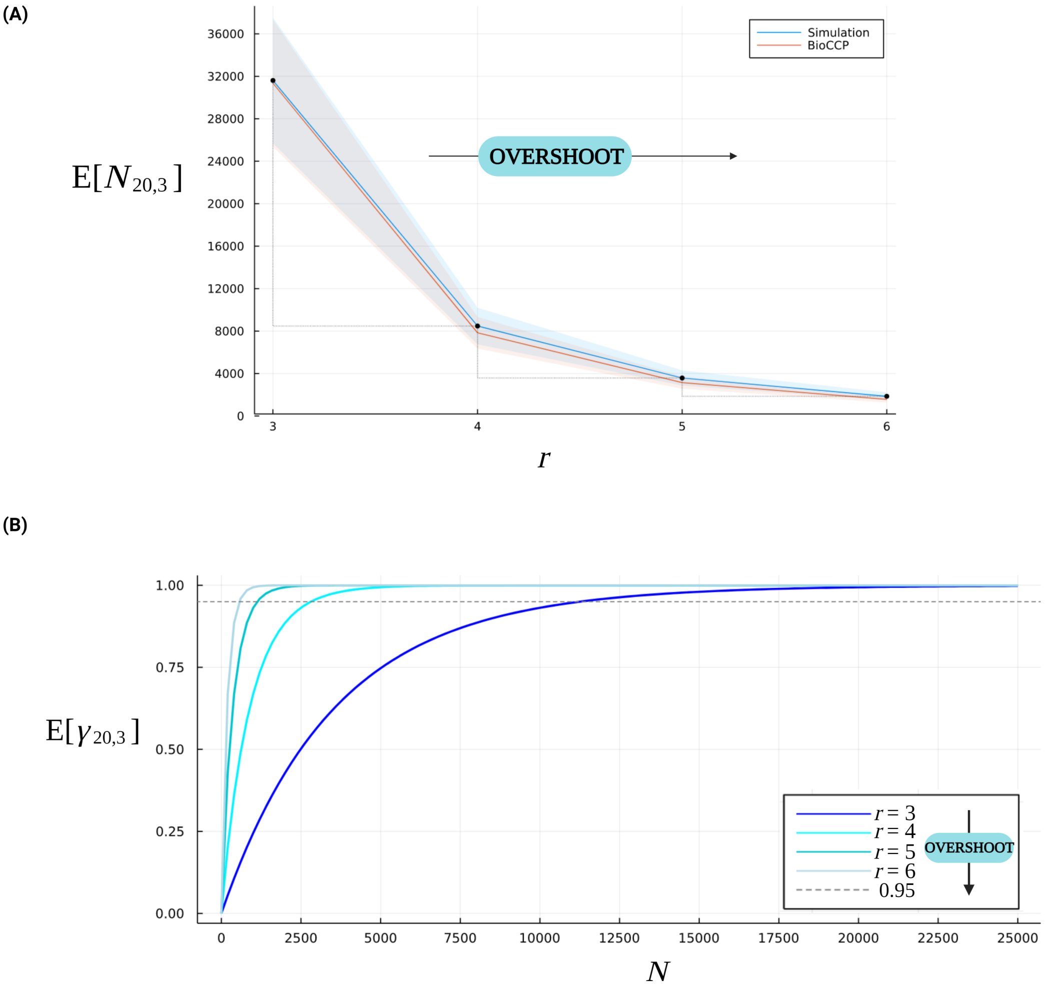

Figure S4. The *Overshoot–Select–Purify* strategy. Consider a multiplex CRISPR/Cas experiment targeting triple combinations of gene knockouts, as specified by the default settings in Table 2 in the manuscript ($k = 3$). **(A)** $N_{20,3}$ decreases as a higher number of r gRNAs are included per construct, exploring a larger fraction of all possible triple combinations of gene knockouts per plant. **(B)** Increased combinatorial coverage $\gamma_{20,3}$ at fixed plant library size N as a result of *Overshoot*.
